# Improving the photovoltage of Cu_2_O photocathodes with dual buffer layers

**DOI:** 10.1038/s41467-023-42799-x

**Published:** 2023-11-09

**Authors:** Jinshui Cheng, Linxiao Wu, Jingshan Luo

**Affiliations:** 1 Institute of Photoelectronic Thin Film Devices and Technology, State Key Laboratory of Photovoltaic Materials and Cells, Key Laboratory of Photoelectronic Thin Film Devices and Technology of Tianjin, Ministry of Education Engineering Research Center of Thin Film Photoelectronic Technology, Renewable Energy Conversion and Storage Center, Nankai University, 300350 Tianjin, China; 2 Frontiers Science Center for New Organic Matter, Nankai University, 300071 Tianjin, China

**Keywords:** Solar fuels, Energy, Photocatalysis

## Abstract

Cuprous oxide (Cu_2_O) is a promising oxide material for photoelectrochemical water splitting (PEC), and increasing its photovoltage is the key to creating efficient overall PEC water-splitting devices. Previous reports are mostly focused on optimizing the energy band alignment between Cu_2_O and the n-type buffer layer to improve the photovoltage of Cu_2_O photocathodes. However, the band alignment between the n-type buffer layer and the protective layer is often ignored. In this work, Cu_2_O photocathodes with a single buffer layer (Ga_2_O_3_) and dual buffer layers (Ga_2_O_3_/ZnGeO_x_) are fabricated, and their PEC performances are compared. Results show that after inserting the second buffer layer (ZnGeO_x_), the onset potential of the Cu_2_O photocathode increases by 0.16 V. Operando electrochemical impedance spectroscopy measurements and analysis of the energy-level diagrams of each layer show that an energy level gradient between Ga_2_O_3_ and TiO_2_ is created when ZnGeO_x_ is introduced, which eliminates the potential barrier at the interface of Ga_2_O_3_/TiO_2_ and improves the photovoltage of the Cu_2_O photocathode. Our work provides an effective approach to improve the photovoltage of photoelectrodes for solar water splitting by introducing dual buffer layers.

## Abstract (web summary)

Increasing the photovoltage of the Cu_2_O photocathode plays an important role in photocatalytic water splitting. Here the authors construct a dual buffer layer to optimize the band alignment between the n-type layer and the protective layer, which improves the onset potential of the Cu_2_O photocathode by 0.16 V.

## Introduction

Solar fuels play a crucial role in the transition from fossil fuel-derived energy to sustainable energy^1–3^. Hydrogen is clean and has a high energy density, these properties make it a key candidate for use as a sustainable fuel^4, 5^. A transition to sustainable hydrogen fuel will contribute greatly toward the development of a low carbon circular economy and achieving net-zero emissions^6^. Photoelectrochemical (PEC) water splitting can solve the problem of solar energy harvest and storage^7^ by converting intermittent solar energy into hydrogen fuel^8,9^, which has received great attention in the past decades^10,11^. Metal oxides are cost-effective materials for producing photoelectrodes to unlock the potential of photoelectrochemical (PEC) water splitting^12,13^.

At present, cuprous oxide (Cu_2_O) is one of the best photocathode materials^14^ and has attracted great interest due to its inherent p-type character^15,16^, natural abundance and low-cost fabrication processes. In addition, its direct bandgap of ~2 eV and appropriate band positions for H_2_ evolution make Cu_2_O a primary choice among low-cost photocathode materials^17^.

It is difficult to achieve unassisted sunlight-driven water splitting using a single Cu_2_O photocathode^18,19^. In general, a self-biased tandem device consisting of the Cu_2_O photocathode and a photoanode or a photovoltaic cell generally needs to be constructed^20,21^. For this tandem system, the key to improving the solar to hydrogen (STH) conversion efficiency is to increase the current density at the point where the *J-V* curves of the photocathode and the photoanode or the photovoltaic cell intersect^22^. Therefore, optimizing the onset potential for the hydrogen evolution reaction (HER) and fill factor of the Cu_2_O photocathode is imperative^20,21^. The onset potential is mainly determined by the photovoltage of Cu_2_O and the overpotential of the HER. A larger photovoltage allows the Cu_2_O photocathode to operate at a more positive bias voltage. When Cu_2_O is in direct contact with the electrolyte, a semiconductor-liquid junction (SCLJ) is created. In this situation, the photovoltage is low (<0.6 V)^23,24^ and photo-corrosion is unavoidable^25,26^. Therefore, a buried solid/solid junction was proposed to further increase the photovoltage of the Cu_2_O photocathode and to inhibit photo-corrosion by preventing it from contacting the electrolyte^27^.

In early reports, TiO_2_ was coupled with Cu_2_O to form a buried junction, but the obtained photovoltage was only 0.46 V^28^, which is mainly due to the limited degree of band-bending of Cu_2_O and the large conduction band offset (△*E*_c_)^29,30^. Following this, the strategy of adding a dipole layer at the interface between Cu_2_O and TiO_2_ was proposed to increase the band-bending of Cu_2_O^31^. However, the improvement in photovoltage was relatively limited. In practice, the photovoltage is determined by the quasi-Fermi levels of the electrons and holes at the interfaces during illumination^29^. Therefore, the n-type semiconductor coupled with Cu_2_O needs to have suitably aligned energy levels^20,30,32^. After continuous efforts, a variety of n-type buffer layers have been developed, such as Al:ZnO^33^, ZnS^24^, GaN^34^, Ga_2_O_3_^20,35,36^, and covalent triazine frameworks (CTF-BTh)^37^, which have successfully increased the photovoltage of the Cu_2_O photocathode from 0.5 V to 1 V (vs. RHE). It is also worth noting that Minami et al. focused on the development of new buffer layers (such as ZnMgO, AlGaO and ZnGeO) and the optimization of the p-n junction, which made an important contribution to improving the photovoltage of the Cu_2_O solar cell^38–41^. In addition, they also emphasized the importance of buffer layer preparation methods^42^.

Indeed, for the Cu_2_O photocathode containing a buried junction, the most ideal solution for improving the photovoltage is to construct a homogeneous Cu_2_O p-n junction, which has an approximately equal electron affinity and reduces interfacial lattice mismatches^43,44^. However, self-compensation makes it challenging to prepare n-type Cu_2_O with excellent photoelectronic properties^30^. Although numerous attempts have been made, the enhancement of photovoltage in the Cu_2_O photocathode is still insufficient^45^.

Moreover, in terms of increasing the photovoltage, the influence of defects and crystal orientation of Cu_2_O cannot be ignored. For example, eliminating interface defects, such as Cu^2+^, has been shown to significantly increase the photovoltage of Cu_2_O-based devices, as it eliminates adverse interfacial defect levels^32,46^. Recently, Niu et al. found that when the Cu_2_O with a high index facet group was used in combination with Ga_2_O_3_, it resulted in a larger photovoltage^47^.

In brief, previous strategies for increasing the photovoltage of Cu_2_O-based devices are mainly focused on improving the band alignment of the p-n junction interface and reducing the defects in Cu_2_O. In essence, it is mainly about optimizing the band alignment of the interface between Cu_2_O and the n-type buffer layer. For a buried junction, after introducing the n-type buffer layer and the protective layer, it is inevitable that an additional interface is formed between the buffer layer and the protective layer. However, the interfacial effects of n-type layers are often ignored. Recently, Moehl et al. found that in addition to the surface properties of Cu_2_O, the interface of Ga_2_O_3_/TiO_2_ also limits the PEC performance of Cu_2_O photocathodes^48^. Therefore, optimizing the band alignment between n-type layers is also important.

In this study, by testing the electrochemical impedance spectroscopy under operando conditions, we found that a potential barrier exists at the interface of Ga_2_O_3_/TiO_2_, which decreases the photovoltage and fill factor of the Cu_2_O photocathode. Next, we inserted a second buffer layer (ZnGeO_x_) between Ga_2_O_3_ and TiO_2_ to form an energy level gradient, which eliminates the potential barrier and improves the band alignment. As a result, the photovoltage of the Cu_2_O photocathode was successfully increased from 0.91 to 1.07 V (vs. RHE). Our work provides an effective approach to improve the photovoltage of photoelectrodes for PEC water splitting.

## Results

### Device structure and characterization

**Fig. 1:**
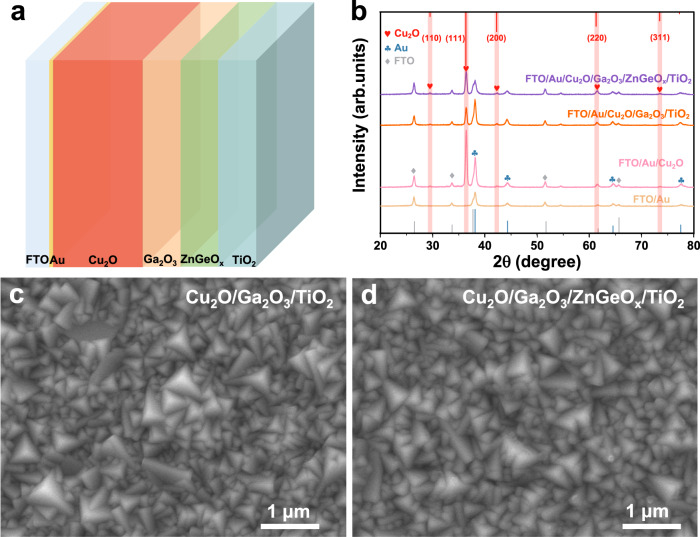
Structure, XRD patterns and SEM imaging of the Cu_2_O photocathodes. **a** A schematic illustration of the structure of the Cu_2_O photocathode with dual buffer layers (Ga_2_O_3_ and ZnGeO_x_). **b** XRD patterns of the different films. The diffraction peaks marked with hearts, clubs and diamonds belong to Cu_2_O, Au and FTO, respectively. **c** Top-view SEM images of the Cu_2_O/Ga_2_O_3_/TiO_2_ photocathode. **d** Top-view SEM images of the Cu_2_O/Ga_2_O_3_/ZnGeO_x_/TiO_2_ photocathode.

To prepare the Cu_2_O photocathode, a Cu_2_O thin film was first electrodeposited onto a gold-coated FTO substrate. Next, the conformal Ga_2_O_3_ and ZnGeO_x_ buffer layers were deposited on the freshly prepared Cu_2_O film by atomic layer deposition (ALD), followed by the deposition of a TiO_2_ protective layer. Finally, the hydrogen evolution catalyst RuO_x_ was deposited by photoelectrochemical deposition (details can be found in the experimental section). The device structure of the Cu_2_O photocathode with dual buffer layers is shown in Fig. 1a. The conformal Ga_2_O_3_ layer directly contacts the Cu_2_O film to form a p-n junction. The control photocathode with a single Ga_2_O_3_ buffer layer (Cu_2_O/Ga_2_O_3_/TiO_2_) is prepared in the same way as that of the photocathode with dual buffer layers (Cu_2_O/Ga_2_O_3_/ZnGeO_x_/TiO_2_), except that it lacks the ZnGeO_x_ layer. The phase and crystal structure of the obtained photocathodes were characterized by X-ray diffraction (XRD). As shown in Fig. 1b, the XRD patterns indicate that the Cu_2_O is a polycrystalline film with an apparent [111] preferential orientation and it has a cubic structure (PDF #05-0667). The diffraction peaks located at 29.5°, 36.4°, 42.3°, 61.3°, and 73.5° correspond to the (110), (111), (200), (220), and (311) crystal planes, respectively. After the deposition of Ga_2_O_3_, ZnGeO_x_ and TiO_2_ layers, no new diffraction peaks were detected, indicating that the Ga_2_O_3_ and ZnGeO_x_ buffer layers and the TiO_2_ protective layer are amorphous.

**Fig. 2:**
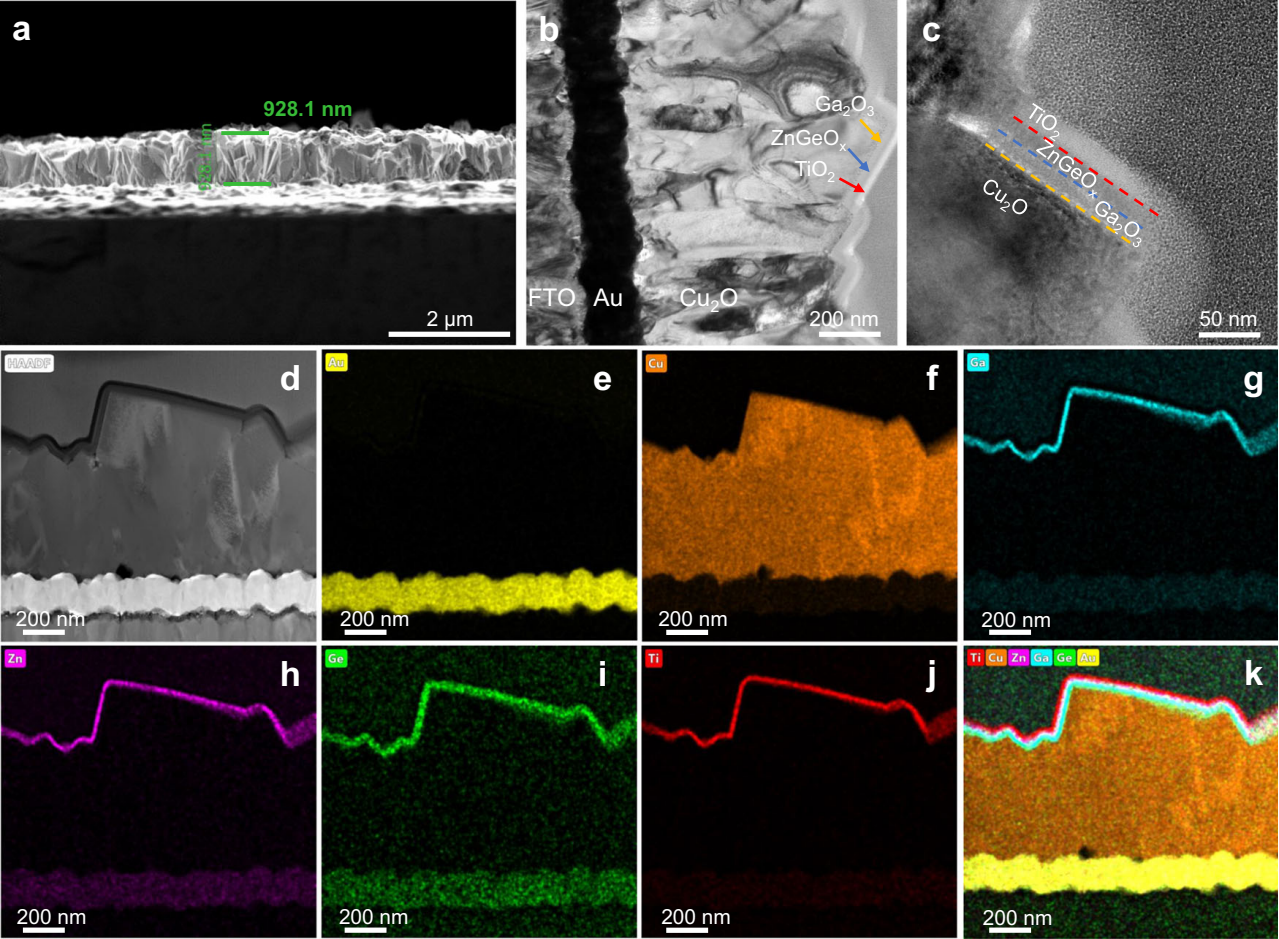
SEM and TEM analysis of the Cu_2_O/Ga_2_O_3_/ZnGeO_x_/TiO_2_ photocathode. **a** Cross-sectional SEM image. **b** Cross-sectional STEM bright-field image. **c** Cross-sectional STEM bright-field image of the top of the Cu_2_O/Ga_2_O_3_/ZnGeO_x_/TiO_2_ photocathode. **d** The STEM high-angle annular dark-field image. **e**–**j** Corresponding elemental mapping images of Au, Cu, Ga, Zn, Ge and Ti, respectively. **k** Combined elemental mapping image of Au, Cu, Ga, Zn, Ge and Ti.

In order to visually observe the morphology, thickness and composition of the photocathodes, scanning electron microscopy (SEM), transmission electron microscopy (TEM) and corresponding elemental mapping were carried out. As shown in Fig. 1c, d, the Cu_2_O/Ga_2_O_3_/TiO_2_ photocathode and the Cu_2_O/Ga_2_O_3_/ZnGeO_x_/TiO_2_ photocathode exhibit similar morphology and grain size. Both are thin films composed of assembled cubic particles with a grain size of approximately 300–500 nm. Meanwhile, from the top-view SEM images, the morphology of the films for the Cu_2_O/Ga_2_O_3_/TiO_2_ photocathode and the Cu_2_O/Ga_2_O_3_/ZnGeO_x_/TiO_2_ photocathode display similar triangular planes, which implies the [111] preferential orientation of Cu_2_O. This result is consistent with the XRD results. The cross-sectional SEM images (Supplementary Fig. 2) clearly show that the thicknesses of the Cu_2_O/Ga_2_O_3_/TiO_2_ photocathode and the Cu_2_O/Ga_2_O_3_/ZnGeO_x_/TiO_2_ photocathode are ~904 nm and ~928 nm, respectively. In order to obtain ZnGeO_x_ films with different thicknesses, we deposited the ZnGeO_x_ film using different numbers of super cycles on clean Si substrates. As shown in Supplementary Fig. 4, the growth rate of ZnGeO_x_ films measured by a step profiler is ~0.55 nm/super-cycle. Therefore, 40 super cycles of ALD result in a ZnGeOx film with a thickness of approximately 22 nm. In addition, as can be observed in the SEM images, after the deposition of the overlayers, the films retain the morphology of Cu_2_O without any aggregation of particles on the surface, indicating that the overlayers cover the Cu_2_O films uniformly and continuously. In order to further examine the heterojunction and multilayer composite structure of the Cu_2_O/Ga_2_O_3_/ZnGeO_x_/TiO_2_ photocathode, TEM and the corresponding elemental mapping were conducted and are depicted in Fig. 2b–k. From the elemental mapping images, Au, Cu, Ga, Zn/Ge, and Ti are detected from bottom to top in the Cu_2_O photocathode, indicating that the device structure is composed of layers. Evidence of the layered structure can also be seen from the line profiles for Ti, Zn, Ge, Ga and Cu elements across the TiO_2_, ZnGeO_x_, and Ga_2_O_3_/Cu_2_O interfaces (Supplementary Fig. 5). The elements in each layer are evenly distributed and in close contact, implying the formation of a close contact buried p-n junction. Furthermore, the uniform distribution of Ge and its position in the same space as Zn indicate that Ge is uniformly doped into ZnO (Fig. 2k). The elemental mapping images further prove that the overlayer coatings are homogeneous and conformal. We conducted the X-ray photoelectron spectroscopy (XPS) measurements to analyze the chemical composition of ZnGeO_x_ films (Supplementary Fig. 6). The atomic ratio of Zn, Ge and O was found to be approximately 21.57:10.64:44.46, which is close to 2:1:4. Therefore, the approximate chemical formula of the ZnGeO_x_ is Zn_2_GeO_4_, and the following ZnGeO_x_ are shorthand for Zn_2_GeO_4_.

### PEC performance and Faradaic efficiency

**Fig. 3:**
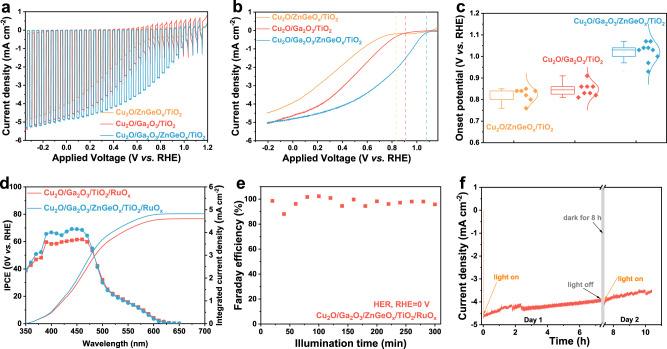
Photoelectrochemical measurements. **a** *J-V* curves of different Cu_2_O-based photocathodes under simulated AM 1.5 G chopped illumination. **b** *J-V* curves of different Cu_2_O-based photocathodes under continuous illumination (AM 1.5 G, 100 mW cm^−2^). **c** Statistical onset potential of different Cu_2_O-based photocathodes. In this plot, boxes represent the interquartile range or the middle half of the values in each group, and the middle lines in each box represent the medians. The lines coming out from each box extend from the maximum to the minimum values of each set; the whiskers show how big a range there is between those two extremes. When data points go above or below 1.5 times the size of the box, they are outliers and they are dotted outside the whiskers. **d** Wavelength-dependent IPCE and the integrated current density of the Cu_2_O photocathodes. **e** The Faradaic efficiency measurement of the Cu_2_O/Ga_2_O_3_/ZnGeO_x_/TiO_2_ photocathode for hydrogen evolution at 0 V_RHE_ under simulated AM 1.5 G irradiation (100 mW cm^−2^). Here, HER stands for hydrogen evolution reaction. RHE stands for reversible hydrogen electrode. **f** The stability measurement of the Cu_2_O/Ga_2_O_3_/ZnGeO_x_/TiO_2_ photocathode at 0 V_RHE_ under simulated AM 1.5 G irradiation (100 mW cm^−2^). All measurements were performed in pH 5.0 phosphate–sulfate electrolyte.

In order to enhance the PEC performance of the Cu_2_O photocathodes, we deposited RuO_x_ as the HER cocatalyst on the surface of the photocathodes using photoelectrochemical deposition. For all the photocathodes, PEC measurements were performed in a phosphate–sulfate buffer electrolyte (pH 5) under simulated AM 1.5 G solar illumination (100 mW cm^−2^) in a standard three-electrode system. Steady-state current-voltage measurements were conducted to evaluate the performance of the Cu_2_O photocathodes. As can be seen in Fig. 3a, the *J-V* curves tested under chopped illumination show rapidly increased current density, which proves the excellent photo response of all the Cu_2_O photocathodes. Meanwhile, the saturated photocurrent density values for the Cu_2_O/Ga_2_O_3_/TiO_2_ and Cu_2_O/Ga_2_O_3_/ZnGeO_x_/TiO_2_ photocathodes are almost the same (5 mA cm^−2^), which is attributed to the same thickness of the Cu_2_O absorber and the same p-n junction interface (Cu_2_O/Ga_2_O_3_). Compared with the Cu_2_O photocathode comprised of a single buffer layer (Cu_2_O/Ga_2_O_3_/TiO_2_), the Cu_2_O photocathode with dual buffer layers (Cu_2_O/Ga_2_O_3_/ZnGeO_x_/TiO_2_) has a slightly higher fill factor.

Here, we define the onset potential as the potential value corresponding to the intercept between the extrapolated tangent lines of the *J-V* curves measured during illumination (AM 1.5 G, 100 mW cm^−2^) and in the dark (Supplementary Fig. 7). In addition, when the bias becomes more negative, the photocurrent density should increase continuously. As shown in Fig. 3b, the onset potential of the Cu_2_O/Ga_2_O_3_/ZnGeO_x_/TiO_2_ photocathode is 1.07 V (RHE), which is positively shifted by 0.16 V (RHE) relative to that of the Cu_2_O/Ga_2_O_3_/TiO_2_ photocathode (0.91 V vs. RHE). The statistical data of the onset potential for different batches of Cu_2_O photocathodes (Fig. 3c) further verifies that inserting the second ZnGeO_x_ buffer layer between the Ga_2_O_3_ layer and the TiO_2_ layer can increase the photovoltage of the Cu_2_O photocathode.

In order to rule out the possibility that the single ZnGeO_x_ buffer layer could improve the onset potential, we also prepared a control photocathode (Cu_2_O/ZnGeO_x_/TiO_2_) with the same Cu_2_O thickness and tested its PEC performance. From the results depicted in Fig. 3a–c, the Cu_2_O/ZnGeO_x_/TiO_2_ photocathode shows a lower saturated photocurrent density (~4.7 mA cm^−2^), a later onset potential (0.84 V vs. RHE) and a poor fill factor. After depositing the dual buffer layers, the total thickness of Ga_2_O_3_ and ZnGeO_x_ is approximately 40 nm. Since the onset potential is relatively sensitive to the thickness of the buffer layer, we also compared the onset potentials of the Cu_2_O/Ga_2_O_3_-40nm/TiO_2_ photocathode and the Cu_2_O/Ga_2_O_3_−20nm/ZnGeO_x_/TiO_2_ photocathode. The results are shown in Supplementary Fig. 8a, b. The onset potential of the Cu_2_O/Ga_2_O_3_−20nm/ZnGeO_x_/TiO_2_ photocathode is still better than that of the Cu_2_O/Ga_2_O_3_-40nm/TiO_2_ photocathode (0.95 V vs. RHE), further proving that the dual buffer layers lead to the improvement of the onset potential.

When comparing the incident photon-to-current conversion efficiency (IPCE) of Cu_2_O photocathodes consisting of a single buffer layer (Ga_2_O_3_) and dual buffer layers (Ga_2_O_3_/ZnGeO_x_), it can be seen that both show similar trends, as shown in Fig. 3d. In the wavelength region from 350 nm to 480 nm, all the Cu_2_O photocathodes have a high quantum efficiency. However, when the wavelength is longer than 480 nm, the IPCE values drop sharply. Furthermore, compared with the Cu_2_O/Ga_2_O_3_/TiO_2_ photocathode, the IPCE of the Cu_2_O/Ga_2_O_3_/ZnGeO_x_/TiO_2_ photocathode is enhanced in the wavelength region from 350 to 480 nm, reaching a maximum of up to 69%.

Stability is an important criterion of Cu_2_O photocathodes. Next, we carefully measured the Faradaic efficiency and the stability of the Cu_2_O/Ga_2_O_3_/ZnGeO_x_/TiO_2_ photocathode. To determine the Faradaic efficiency for hydrogen evolution, PEC water-splitting experiments were carried out in a gas-tight H-cell under continuous illumination (AM 1.5 G, 0 V vs. RHE) and the generated hydrogen was measured using an online gas chromatograph. As shown in Fig. 3e, although the values of the Faradaic efficiency fluctuate due to the irregular release of bubbles and small cell headspace, the average Faraday efficiency is close to 100%, indicating that the photogenerated electrons from Cu_2_O photocathode are all used to reduce protons to produce hydrogen. Figure 3f shows the photocurrent density versus time plots under continuous illumination (0 V vs. RHE). The Cu_2_O photocathode with dual buffer layers showed excellent stability over 10 h. The decrease and fluctuation of the photocurrent density in the first 3 h are mainly caused by the attached bubbles on the surface of the Cu_2_O photocathode.

### Deducing the mechanism responsible for the improvement in onset potential

After proving that dual buffer layers can improve the HER onset potential, it is important to identify the underlying mechanism. The HER onset potential is determined by the photovoltage and overpotential. Since the same HER catalyst (RuO_x_) and the same electrolyte were used to evaluate the PEC performance of all the Cu_2_O photocathodes, the improvement in the onset potential was mainly due to the increased photovoltage rather than the improvement in overpotential. Moreover, the open-circuit potential curves (Supplementary Fig. 10) show that the open-circuit potential difference of the Cu_2_O/Ga_2_O_3_/ZnGeO_x_/TiO_2_/RuO_x_ photocathode (620 mV) is larger than that of the Cu_2_O/Ga_2_O_3_/TiO_2_/RuO_x_ photocathode (450 mV) and the Cu_2_O/ZnGeO_x_/TiO_2_/RuO_x_ photocathode (390 mV), which further suggests that the Cu_2_O/Ga_2_O_3_/ZnGeO_x_/TiO_2_/RuO_x_ photocathode possesses an enhanced charge carrier separation efficiency and an increased photovoltage. The photovoltage is related to the defects in Cu_2_O (bulk defects and interfacial defects) and the band alignment optimization of the Cu_2_O photocathode. However, both the Cu_2_O/Ga_2_O_3_/ZnGeO_x_/TiO_2_ and the Cu_2_O/Ga_2_O_3_/TiO_2_ photocathodes adopt the same Cu_2_O absorber layer, and the ZnGeO_x_ layer has no direct contact with the Cu_2_O film or RuO_x_. As a result, the effect of: (1) defects in Cu_2_O and (2) the Cu_2_O/Ga_2_O_3_ p-n junction on the optimization of photovoltage are excluded. The only remaining possible cause is that the insertion of the ZnGeO_x_ layer improves the band alignment between Ga_2_O_3_ and TiO_2_. We speculate that the ZnGeO_x_ layer provides an energy level gradient between Ga_2_O_3_ and TiO_2_, which facilitates the transport of photogenerated electrons and increases the photovoltage of the Cu_2_O photocathode.

**Fig. 4:**
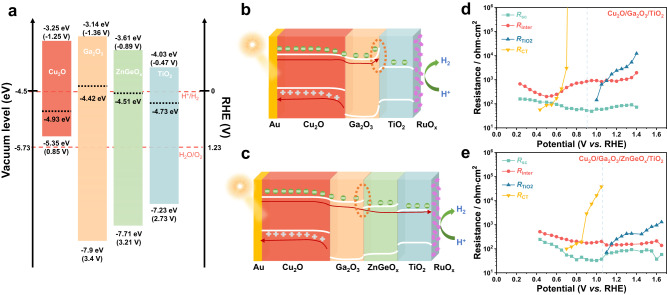
Energy diagrams and extracted resistance. **a** Energy diagrams of Cu_2_O, Ga_2_O_3_, ZnGeO_x_, and TiO_2_ (from left to right) derived from Kelvin probe force microscopy (KPFM), XPS valence-band spectra and the Tauc plots. **b** Schematic diagram of the photogenerated carrier transport behavior in the Cu_2_O/Ga_2_O_3_/TiO_2_/RuO_x_ photocathode under illumination. **c** Schematic diagram of the photogenerated carrier transport behavior in the Cu_2_O/Ga_2_O_3_/ZnGeO_x_/TiO_2_/RuO_x_ photocathode under illumination. **d** Resistances from the EIS fitting of the Cu_2_O/Ga_2_O_3_/TiO_2_/RuO_x_ photocathode under illumination. **e** Resistances from the EIS fitting of the Cu_2_O/Ga_2_O_3_/ZnGeO_x_/TiO_2_/RuO_x_ photocathode under illumination.

To confirm our speculation, Fermi levels, valence-band levels and the optical band gaps of each layer in the Cu_2_O/Ga_2_O_3_/ZnGeO_x_/TiO_2_ photocathode were determined using Kelvin probe force microscopy (KPFM), XPS valence-band spectra and Tauc plots (Supplementary Figs. 11–13). Specifically, Au with a known work function of 5.1 eV was used to calibrate the work function of the KPFM tip for a clean and conductive surface. Since the contact potential difference (CPD) between the sample and the KPFM tip corresponds to their Fermi level (*E*_F_) difference, the difference in *E*_F_ between the sample and Au can be obtained by measuring their CPD. Next, the difference between the valence-band maximum (*E*_VBM_) and *E*_F_ can be obtained from the XPS valence spectra and thus we can determine the *E*_VBM_. The conduction-band minimum of the samples can also be deduced based on their band gaps. Detailed calculation methods are shown in the experimental section. Finally, the energy-level diagrams of Cu_2_O, Ga_2_O_3_, ZnGeO_x_ and TiO_2_ are constructed and shown in Fig. 4a. The Fermi level of ZnGeO_x_ is almost identical to that of Ga_2_O_3_. However, its conduction band minimum lies within that of Ga_2_O_3_ and TiO_2_. Therefore, the second buffer layer (ZnGeO_x_) does provide an energy level gradient between the Ga_2_O_3_ and TiO_2_ in the Cu_2_O photocathode.

In order to shed light on how the energy level gradients optimize the band alignment and improve the photovoltage, a powerful non-destructive characterization method is necessary to reveal the underlying transport behavior of the photogenerated electrons through the interface of each layer in the photocathode under operando condition. Recently, Moehl et al. established a resistance-based method using electrochemical impedance spectroscopy (EIS) to identify relevant charge carrier transfer processes under operation^44^. Under illumination, the minority charge carriers dominate the light-induced processes. They investigated the underlying potential-dependent processes during water splitting for the Cu_2_O/Ga_2_O_3_/TiO_2_ photocathode and assigned the resistance observed in the EIS spectra under illumination to the relevant charge carrier transfer process, denoted as *R*_CT_, *R*_SC_, *R*_TiO2_ and *R*_inter_. Moreover, Moehl et al. concluded that the main limitations of the onset potential for the Cu_2_O/Ga_2_O_3_/TiO_2_ photocathode are the adverse barriers present at the Ga_2_O_3_/TiO_2_ and TiO_2_/RuO_x_ interface^44^.

In order to reveal the underlying transport behavior of the photogenerated electrons through the interface of each layer in the Cu_2_O photocathode, we compared the electrochemical impedance spectra of Cu_2_O/Ga_2_O_3_/TiO_2_ and Cu_2_O/Ga_2_O_3_/ZnGeO_x_/TiO_2_ photocathodes, the measurements were conducted under illumination (~120 W m^−2^). The Nyquist plots of both Cu_2_O photocathodes are displayed in Supplementary Fig. 15b–g. The corresponding equivalent circuit model used to fit the Cu_2_O devices is shown in Supplementary Fig. 15a. The resistances of *R*_CT_, *R*_SC_, *R*_TiO2_ and *R*_inter_ from the EIS fitting procedure were extracted (Fig. 4d, e). All resistances are present during water splitting. In detail, *R*_CT_, the low-frequency resistance, is related to the hydrogen-generating charge transfer over the hydrogen-evolving catalyst (HEC) into the electrolyte solution, implying that Ru(IV) is reduced to Ru(III). *R*_CT_ is related to the current flow for the reduction step of RuO_x_. When the bias is more negative than the onset potential for HER, more photogenerated charge carriers reach the electrolyte through RuO_x_. RuO_x_ will be reduced faster, and the *R*_CT_ will decrease dramatically. *R*_SC_ is related to the recombination process of the photogenerated charge carriers. It represents the recombination resistance of the photogenerated electron-hole pairs. Before the bias reaches the onset potential for HER, the electrons flow inside the Cu_2_O photocathode is negligible. Therefore, the *R*_SC_ is normally constant. However, once the recombination of photogenerated carriers is suppressed, such as when the bias exceeds the onset potential, *R*_SC_ will increase. *R*_TiO2_ is related to a resistive limitation at the TiO_2_/RuO_x_ interface. This resistance is caused by the unfavorable band bending for the contact between TiO_2_ and RuO_x_^44^. Finally, *R*_inter_ is related to the interfacial defects of Cu_2_O and the interfacial barrier of Ga_2_O_3_/TiO_2_ caused by the upward band bending of the conduction band of Ga_2_O_3_ (Fig. 4b).

As shown in Fig. 4d, e, the *R*_TiO2_ values for Cu_2_O/Ga_2_O_3_/TiO_2_ and Cu_2_O/Ga_2_O_3_/ZnGeO_x_/TiO_2_ photocathodes both show an exponential trend in reduction in two segments, indicating the increasing current flow for the reduction of the RuO_x_ catalysts. Before H_2_ evolution proceeds, their *R*_SC_ is almost constant, implying that the flow of electrons inside the Cu_2_O photocathode is negligible. When the reverse bias exceeds the HER onset potential, both of their *R*_SC_ values increase gradually, which is due to the suppressed recombination of the photogenerated carriers. Furthermore, for the Cu_2_O/Ga_2_O_3_/ZnGeO_x_/TiO_2_ photocathode, the increasing slope of its *R*_SC_ is larger than that of the Cu_2_O/Ga_2_O_3_/TiO_2_ photocathode, indicating that the Cu_2_O/Ga_2_O_3_/ZnGeO_x_/TiO_2_ photocathode has a better fill factor. This is consistent with the results of the measured *J-V* curves (Fig. 3a, b).

As for the *R*_CT_ of the Cu_2_O photocathodes with a single buffer layer (Ga_2_O_3_) and dual buffer layers (Ga_2_O_3_/ZnGeO_x_), it shows a steep downward trend, indicating that Ru (VI) is rapidly reduced to Ru(III). The trends observed here for the changes in *R*_CT_, *R*_SC_, and *R*_TiO2_ are consistent with the results presented by Moehl et al.^44^. However, for Cu_2_O/Ga_2_O_3_/TiO_2_ and Cu_2_O/Ga_2_O_3_/ZnGeO_x_/TiO_2_ photocathodes, their *R*_inter_ values show different trends. Both of their *R*_inter_ values are almost constant before H_2_ evolution begins, and the *R*_inter_ of the Cu_2_O/Ga_2_O_3_/ZnGeO_x_/TiO_2_ photocathode is smaller than that of the Cu_2_O/Ga_2_O_3_/TiO_2_ photocathode. After the reverse bias exceeds the HER onset potential, the *R*_inter_ of the Cu_2_O/Ga_2_O_3_/TiO_2_ photocathode decreases initially and then increases gradually. However, the *R*_inter_ of the Cu_2_O/Ga_2_O_3_/ZnGeO_x_/TiO_2_ photocathode shows a gradually increasing trend without decreasing. According to the analysis results of Moehl et al., the decrease in *R*_inter_ after passing the HER onset potential is caused by the interfacial barrier of Ga_2_O_3_/TiO_2_^44^. However, when the reverse bias is increased further, *R*_inter_ will increase again. This process represents the reduced recombination of photogenerated charge carriers. The increase of *R*_inter_ is caused by the decreasing interfacial barrier of Ga_2_O_3_/TiO_2_, since the increasing reverse bias elevates the conduction band of Ga_2_O_3_ and inhibits the upward band bending of Ga_2_O_3_.

As for the *R*_inter_ trend for the Cu_2_O/Ga_2_O_3_/ZnGeO_x_/TiO_2_ photocathode, the decreasing trend disappears. Therefore, it is reasonable to consider that the interfacial barrier in Ga_2_O_3_/TiO_2_ is eliminated. As shown in Fig. 4a, since the Fermi levels of Ga_2_O_3_ and ZnGeO_x_ are similar, the upward band bending of Ga_2_O_3_ is suppressed (Fig. 4c). As the conduction band level of ZnGeO_x_ lies between that of Ga_2_O_3_ and TiO_2_, the insertion of ZnGeO_x_ provides an energy level gradient (Fig. 4c), improving the band alignment of the Cu_2_O photocathode. As a result, the photogenerated electrons can smoothly transport from Cu_2_O to the RuO_x_ catalysts without any barriers, reducing the interfacial energy loss. Finally, the influence on the *J-V* curves is shown by the positive shift of the onset potential and the increased fill factor.

In summary, here, we demonstrate that inserting a second buffer layer (ZnGeO_x_) between the Ga_2_O_3_ layer and the TiO_2_ layer can improve the onset potential of the Cu_2_O photocathode by 0.16 V. By combining the energy-level diagrams of each layer in the Cu_2_O photocathode with the results obtained from operando electrochemical impedance spectroscopy measurements, we found that the insertion of ZnGeO_x_ introduces an energy level gradient and eliminates the interfacial barrier of Ga_2_O_3_/TiO_2_. As a result, it improves the band alignment and increases the photovoltage of the Cu_2_O photocathode. Our work provides an effective approach to increase the photovoltage of photoelectrodes with buried junctions.

## Discussion

In this work, we have shown that the construction of dual buffer layers in the Cu_2_O photocathode can increase the photovoltage to 1.07 V. However, given that the bandgap of Cu_2_O is 2 eV, the theoretical maximum photovoltage of the Cu_2_O photocathode can reach 1.6 V^49^, which leaves a lot of space for further improvement of its photovoltage.

In addition to optimizing the band alignment between the n-type buffer layer and the protective layer, it is important to optimize the band alignment of the p-n junction interface. For transparent n-type layers coupled with Cu_2_O, there should be a larger Fermi level difference and a smaller conduction band offset relative to Cu_2_O. In addition, n-typer layers should have excellent electrical conductivity. Up until now, Ga_2_O_3_ is still one of the best n-type buffer layers for the Cu_2_O photocathode. However, insulator-like Ga_2_O_3_ has a larger resistance, which is not conductive to the transport of charge carriers. Increasing the carrier densities and conductivity of Ga_2_O_3_ by heteroatom doping may be an effective solution^32^. In addition, the influence of interfacial defects or surface states on the photovoltage of the Cu_2_O photocathode cannot be ignored. These defects, such as Cu^0^ or Cu^2+^, can narrow the splitting of the quasi-Fermi level and trigger the recombination of hole-electron pairs by restricting the Fermi level of Cu_2_O, which eventually decreases the photovoltage^29,50^. However, the current understanding of defect generation and the specific mechanism affecting the photovoltage is unclear and further investigation is required. The preparation of high-quality single-crystal Cu_2_O is also worth further development and exploration. Finally, the effect of different crystal groups on the photovoltage of Cu_2_O photocathodes is also a very interesting and important research point.

## Methods

### Chemicals

All the chemicals and reagents were used as purchased without further purification. Chemicals and reagents used in this work are as follows: copper (II) sulfate (≥99.95%, Sigma-Aldrich), DL-Lactic acid (90%, TCI), potassium phosphate dibasic anhydrous (99.99%, Aladdin), potassium hydroxide (99.999%, Aladdin), bis(μ-dimethylamino)tetrakis(dimethylamino)digallium (98%, Strem Chemicals), tetrakis-dimethylamino titanium (99%, Strem Chemicals), diethyl zinc (99.99%, Dongguan NanoFrontier Microelectronic Equipment Co. Ltd), tetramethoxygermane (98%, Alfa Aesar), sodium sulfate anhydrous (99%, Aladdin), potassium dihydrogen phosphate (99.5%, Aladdin), TritonX-100 (*n* ≈ 10, Aladdin). Ultrapure water (18.2 MΩ⋅cm, Purelab Ultra, ELGA) was used for the preparation of aqueous solutions.

### Electrodeposition of cuprous oxide (Cu_2_O)

All Cu_2_O photocathodes were prepared using FTO coated with a 160 nm Au film as the substrates. In order to enhance the adhesion between the Au film and FTO, a 20 nm Cr film was inserted between them. The Au film and the Cr film were prepared by DC magnetron sputtering. The electroplating solution was a basic solution of lactate-stabilized copper sulfate prepared by dissolving 4 g CuSO_4_, 33.75 g lactic acid and 10.885 g K_2_HPO_4_ in 125 mL H_2_O. A 2 M KOH solution was then used to adjust the solution pH to 12. The electrodeposition of Cu_2_O was performed using galvanostatic mode with a current density of −0.1 mA cm^−2^ in a two-electrode configuration. The electrodeposition time of all Cu_2_O films was 100 min, which resulted in a Cu_2_O film with a thickness of approximately 860 nm. A large piece of frosted glass coated with a thicker Au film was used as the counter electrode. During deposition, the electrolyte was maintained at 30 °C using a hot plate fitted with an in-situ temperature probe. Immediately after the electrodeposition, the surface of the Cu_2_O film was washed with plenty of water and dried with N_2_.

### Atomic layer deposition of overlayers

For all the Cu_2_O photocathodes, all freshly prepared Cu_2_O films were immediately transferred to the atomic layer deposition chamber for subsequent film deposition after being dried with N_2_. A 3 × 7 cm Cu_2_O film substrate was cut into two pieces and put into the same deposition chamber. The ALD-Ga_2_O_3_ film was then deposited onto the two Cu_2_O film samples. One sample was then removed and an ALD-ZnGeO_x_ film was deposited on the other sample. Finally, the ALD-TiO_2_ protective layer was deposited on the two samples in the same batch to obtain the Cu_2_O/Ga_2_O_3_/TiO_2_ device and the Cu_2_O/Ga_2_O_3_/ZnGeO_x_/TiO_2_ device, respectively. As for the preparation of the Cu_2_O/ZnGeO_x_/TiO_2_ device, the ALD-ZnGeO_x_ film and ALD-TiO_2_ film were directly deposited on the freshly prepared Cu_2_O film in turn. A portion of the exposed gold substrate was masked with Kapton tape prior to the deposition of the overlayers. The ALD-Ga_2_O_3_ film was deposited by running 200 periodic cycles consisting of 1 cycle of bis(μ-dimethylamino)tetrakis(dimethylamino)digallium and water, at 150 °C, which gives a film of approximately 20 nm in thickness. In order to ensure that there was a sufficient amount of Ga in the deposition cavity, the Ga source cylinder was heated at 124 °C. During deposition, the Ga precursor ALD valve was opened for 0.25 s, followed by a 20 s N_2_ purge. Germanium-doped zinc oxide (ZnGeO_x_) was deposited by running 40 super cycles consisting of 1 cycle of tetramethoxygermane and water after 3 cycles of diethyl zinc and water, at 120 °C, which gives a film of approximately 20 nm in thickness. The Ge precursor ALD valve was opened for 0.18 s, followed by a 15 s N_2_ purge. Next, a 0.1 s pulse of H_2_O was used, followed by a 20 s N_2_ purge. The Zn precursor ALD valve was opened for 0.07 s, followed by a 15 s N_2_ purge. Then a 0.1 s pulse of H_2_O was used, followed by a 20 s N_2_ purge. Titanium dioxide (TiO_2_) was deposited at 150 °C using tetrakis-dimethylamino titanium (TDMAT) and H_2_O as the Ti and O precursors, respectively. The growth rate of the ALD-TiO_2_ film was 0.056 nm/cycle. To ensure that the vapor pressure was sufficient, TDMAT was heated to 80 °C. The precursor temperatures of Ge, Zn, and O were kept at room temperature. The ALD process was operated in a thermal ALD system (NCE-200R). For scanning voltammetry testing and the measurement of open-circuit potential, the thickness of the TiO_2_ protective layer was 20 nm, while for stability testing and the Faraday efficiency measurements, the thickness of the TiO_2_ protective layer was 120 nm.

### RuO_x_ catalyst photoelectrodeposition

Galvanostatic photoelectrodeposition was used to deposit the RuO_x_ catalyst onto the Cu_2_O samples. Typically, RuO_x_ was deposited at a current density of −30 μA cm^−2^ for 6 min under simulated one sun illumination in a standard three-electrode configuration. The electroplating solution was prepared by dissolving 2.6 mg KRuO_4_ in 10 mL H_2_O.

### Material characterization

XRD measurements were performed on a Rigaku X-ray diffractometer using Cu Kα radiation, a scanning range of 2θ = 20–80° and a scan rate of 10° min^−1^. The morphology of the films was characterized using a high-resolution scanning electron microscope (Apreo S LoVac). For TEM imaging, the cross-sectional lamellae of the Cu_2_O photocathode samples were prepared using the focused ion beam technique (Helios NanoLab 460HP). Pt deposition was used to protect the surface. TEM characterization was performed on a TEM (FEI-Talos F200X) operating at 200 kV. Both TEM and STEM modes were used. EDS mapping was acquired by using quadrant EDS detectors in STEM mode. The composition of the ALD-ZnGeOx film was analyzed by X-ray photoelectron spectroscopy (Thermo Fisher Scientific ESCALAB 250Xi). Optical absorption spectra were collected at room temperature with a UV-vis spectrophotometer (UV-2600, Shimadzu). The growth rate of the ALD-ZnGeO_x_ film was tested using a step profiler (Kosaka ET200). Measurements of the XPS valence-band spectra were conducted using a photoelectron spectrometer (Thermo Fisher Scientific ESCALAB 250Xi) featuring monochromatic Al Kα radiation (hν = 1486.68 eV) under a pressure of 1 × 10^−9^ mbar. The Fermi level edge of the Au reference film was used to calibrate the binding energy scale. The contact potential difference (CPD) and KPFM images were obtained in an ambient atmosphere using a Bruker Dimension Icon instrument. The CPD between the sample films and the KPFM tip (conductive CoCr-coated Sb-doped Si) can be determined using the following equations^51–53^:

1 $$-e\times{V}_{{{{{{\rm{CPD}}}}}}}={W}_{{{{{{\rm{Tip}}}}}}}-{W}_{{{{{{\rm{Film}}}}}}}$$

2 $${E}_{{{{{{\rm{fFilm}}}}}}}=-{W}_{{{{{{\rm{Film}}}}}}}$$

Where *W*_Tip_ and *W*_Film_ are the work functions of the KPFM tip and the as-obtained film, respectively, and *E*_fFilm_ is the Fermi level of the as-obtained film. In order to determine the Fermi level of the sample films, Au with a known work function of 5.1 eV was used to calibrate the work function of the KPFM tip for a clean and conductive surface. The valence-band maximum (*E*_VBM_) can be determined using the following equation:

3 $${E}_{{{{{{\rm{VBM}}}}}}}={E}_{{{{{{\rm{fFilm}}}}}}}-{E}_{{{{{{\rm{edge}}}}}}}$$

Where *E*_edge_ is the valence-band edge. *E*_edge_ can be obtained via extrapolation to the linear part of the binding-energy edge in the XPS valence-band spectra. It represents the difference between *E*_VBM_ and *E*_fFilm_, for each material.

### Photoelectrochemical analysis

Current-voltage measurements were conducted in homemade PEEK cells using a three-electrode configuration, where a Cu_2_O photocathode was the working electrode, Pt plate is the counter electrode and Ag/AgCl/sat. KCl was the reference electrode. A scan rate of 10 mV s^−1^ in the cathodic direction was used to acquire the data. The electrolyte solution was a pH 5 buffer solution containing 0.5 M Na_2_SO_4_ and 0.1 M sodium phosphate. The photoelectrochemical performance of the photocathodes was studied using a CHI-760E electrochemical workstation. Specifically, the photo response of the photocathodes was measured under simulated AM 1.5 G illumination (100 mW cm^−2^) generated from a Xe-lamp (MC-X301B) equipped with an AM 1.5 G filter. The light intensity was controlled by light path distance which is determined by measuring the short-circuit current of a calibrated silicon diode with a KG 3 filter. All potentials have been referenced to the RHE using the following equations:

4 $${V}_{{{{{{\rm{RHE}}}}}}}={V}_{{{{{{\rm{Ag}}}}}}/{{{{{\rm{AgCl}}}}}}}+0.197\,{{{{{\rm{V}}}}}}+0.059\,{{{{{\rm{V}}}}}}\times {{{{{\rm{pH}}}}}}$$

IPCE was measured in a home-built system equipped with a Xe-lamp and a monochromator. Measurements were conducted using a three-electrode configuration at 0 V versus the reversible hydrogen electrode (RHE). Comparison with a calibrated Si photodiode allowed the calculation of the IPCE.

### Stability and Faradaic efficiency measurement

For the stability tests, the Cu_2_O photocathode containing a 120 nm TiO_2_ film was inserted in a quartz cell filled with electrolyte, and the change in behavior of the photocurrent density over time was monitored under a constant bias voltage of 0 V (vs. RHE). The measurement was conducted under rapid stirring in a standard three-electrode configuration. The light intensity was calibrated to 100 mW cm^−2^. The electrolyte solution was a pH 5 buffer solution, containing 0.5 M Na_2_SO_4_ and 0.1 M sodium phosphate.

The Faradaic efficiency of the photocathode was measured in a gas-tight photoelectrochemical H-cell under a constant bias voltage of 0 V (vs. RHE), equipped with an anion exchange membrane (Selemion AMVN, AGC Inc.) separating both compartments and with a quartz window (2 cm in diameter). An Ag/AgCl (KCl sat.) reference electrode was employed and a platinum mesh was used as the counter electrode. Ar gas was bubbled through the catholyte at a flow rate of 15 mL min^−1^ during the measurement. The resulting gas products were passed through the sample loop of a gas chromatograph equipped with a thermal conductivity detector (TCD) and analysis was carried out in 20-min intervals. The light intensity was calibrated to 100 mW cm^−2^. The electrolyte solution was a pH 5 buffer solution, containing 0.5 M Na_2_SO_4_ and 0.1 M sodium phosphate. The measured amount of hydrogen produced, in moles, was compared to the observed photocurrent density, which provided the Faradaic efficiency.

### Electrochemical impedance measurements

The electrochemical impedance spectroscopy (EIS) measurements were carried out following a previously reported method^48^. In brief, EIS measurements were carried out under illumination (~120 W m^−2^) using a three-electrode configuration in pH 5.0 phosphate–sulfate electrolyte. In order to minimize the size of bubbles formed, 1 mM TritonX was dissolved in the electrolyte. Full impedance spectra were measured using an Autolab M204 at frequencies from 1 MHz to 0.2 Hz. The range of the bias potential was 1.65–0.2 V (vs. RHE). The bias potential steps were 50 mV and the equilibration time at each bias potential step was normally 30 s. The EIS spectra were fitted with Zview. For details about the model used for the fitting procedure, please see below (Supplementary Fig. 15a). Before each EIS measurement, the device was preconditioned at the starting potential for 180 s to achieve the steady state of the RuO_x_/electrolyte solution interface.

Based on the number of processes observed in the Nyquist plot, a corresponding number of simple resistors and capacitors are used to fit the EIS spectra. Resistors and capacitors corresponding to the same process are connected in parallel, while elements of different processes are connected in series (Supplementary Fig. 15a). To account for the non-ideality of the capacitors constant phase elements, CPEs, have been used (with the exponent accounting for the ideality of the CPE not going below 0.8). This fitting method could result in a less accurate overview of the photophysical processes. However, the determined resistances and their dependence on the applied potential still enable us to draw valuable conclusions on the operation of the system and the assigning of the resistances to certain photophysical or electrochemical processes. Generally, we can assume that photogenerated charge carriers are subjected to a recombination process inside the space charge region of the photoabsorber. This process normally takes place in the μs range for sufficiently efficient devices and is therefore situated in the HF region of the EIS measurements (MHz down to KHz). The recombination resistance associated with this process will increase when the recombination current is suppressed. Therefore, before the bias reaches the HER onset potential or the recombination is suppressed by strong band bending, the recombination resistance associated with this process is normally constant. As for the slow process associated with the electron charge transfer into the electrolyte, the corresponding resistance in the EIS measurements appears in the mHz to Hz range^48^. The characteristic frequency of each resistance element is displayed in Supplementary Table 1.

### Supplementary information

Supplementary Information

Peer Review File

## Data Availability

The datasets generated during and/or analyzed during the current study are available in the figshare repository, 10.6084/m9.figshare.24050985.
