# Supplementary material for: Improving the photovoltage of Cu2O photocathodes with dual buffer layers
Source: Nat Commun. 2023 Nov 9;14:7228. doi: 10.1038/s41467-023-42799-x (PMC10636130; doi:10.1038/s41467-023-42799-x)
Supplement: Supplementary file 1 — Supplementary Information [file 41467_2023_42799_MOESM1_ESM.pdf]

Supplementary Information

Improving the Photovoltage of Cu_2O Photocathodes with Dual Buffer Layers

Jinshui Cheng[1], Linxiao Wu[1], Jingshan Luo[1]*

[1]Institute of Photoelectronic Thin Film Devices and Technology, Solar Energy Research Center, Key Laboratory of Photoelectronic Thin Film Devices and Technology of Tianjin, Ministry of Education Engineering Research Center of Thin Film Photoelectronic Technology, Renewable Energy Conversion and Storage Center, Nankai University, 300350 Tianjin, China

[2]Frontiers Science Center for New Organic Matter, Nankai University, 300071 Tianjin, China

*Email: jingshan.luo@nankai.edu.cn

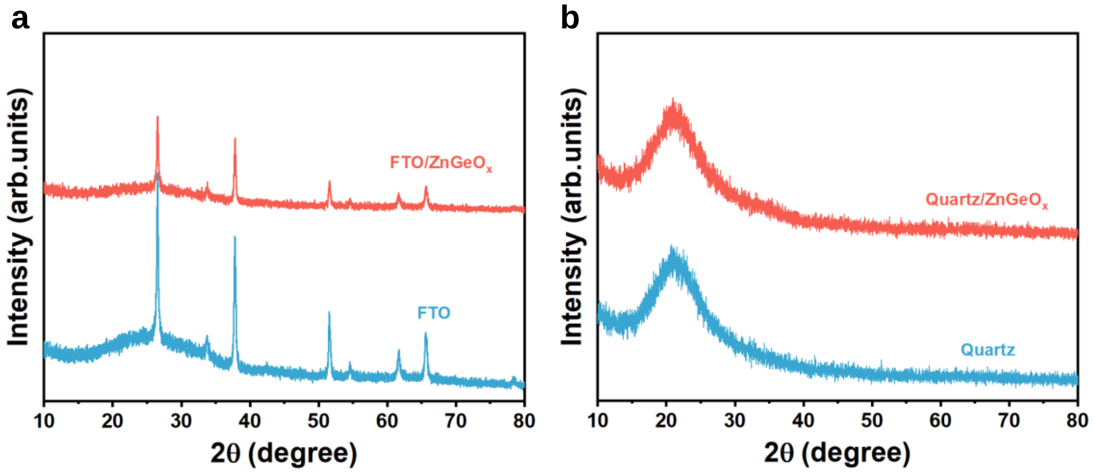

Supplementary Figure 1. XRD patterns of the ALD-ZnGeO$_x$ films. a XRD patterns of the ALD-ZnGeO$_x$ films deposited on FTO substrate. **b** XRD patterns of the ALD-ZnGeO$_x$ films deposited on quartz substrate.

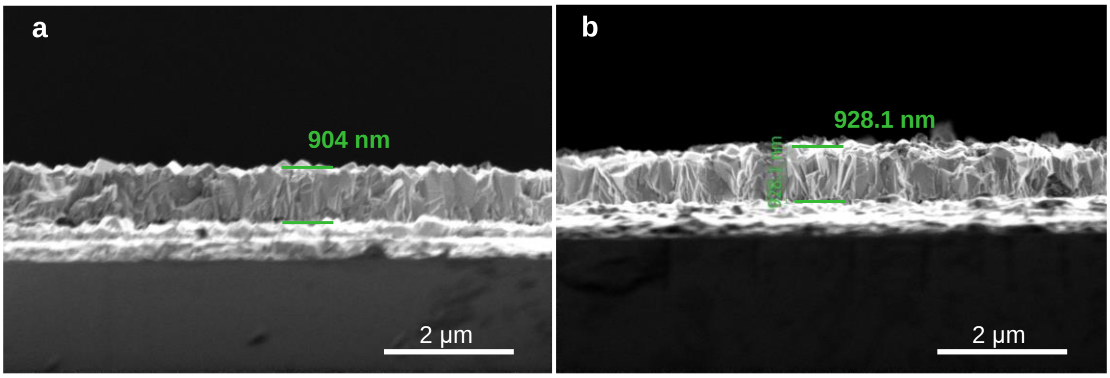

Supplementary Figure 2. Cross-sectional SEM images. **a** Cross-sectional SEM images of the $Cu_2O/Ga_2O_3/TiO_2$. **b** Cross-sectional SEM images of the $Cu_2O/Ga_2O_3/ZnGeO_x/TiO_2$ photocathodes.

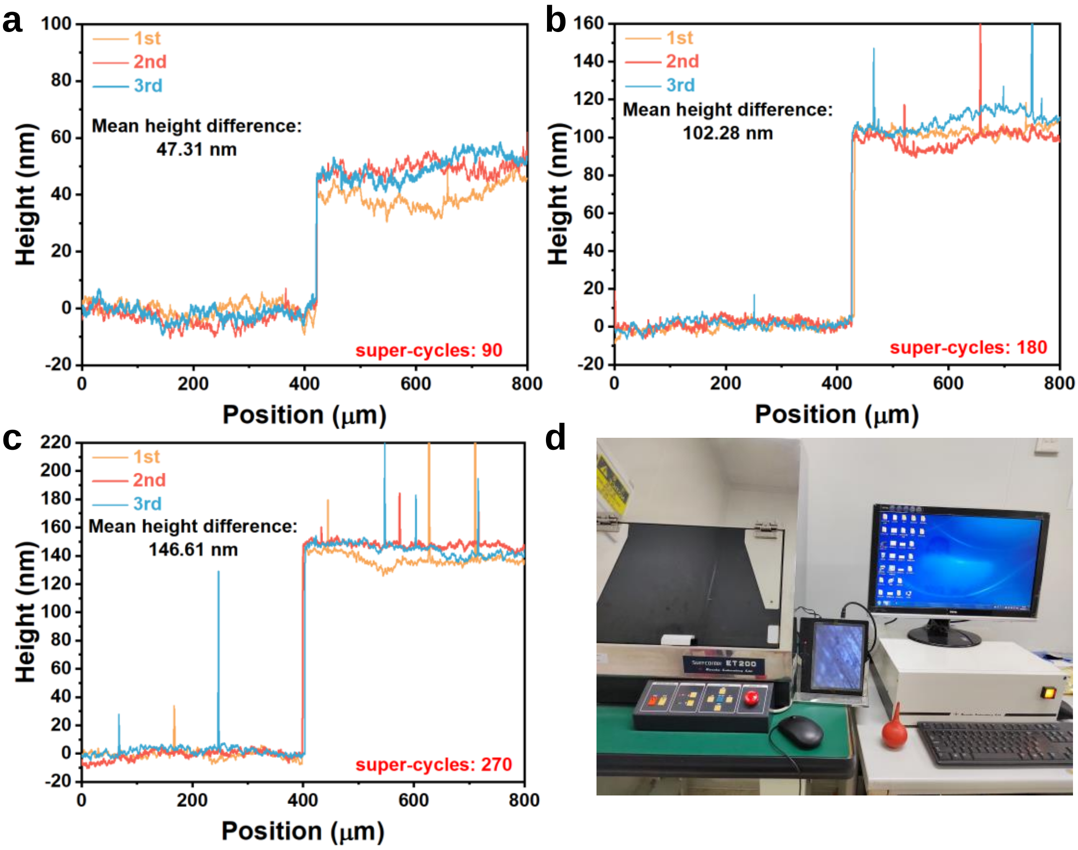

Supplementary Figure 3. Thickness testing of $ZnGeO_x$ films. The original data for the thickness of ALD-$ZnGeO_x$ with **a**, 90 super-cycles, **b**, 180 super-cycles, **c**, 270 super-cycles, which were measured using a step profiler. **d** Photo of the step profiler. The $ZnGeO_x$ films were deposited on clean Si substrates. The thickness of each $ZnGeO_x$ sample was tested three times and its average was taken to fit the growth rate.

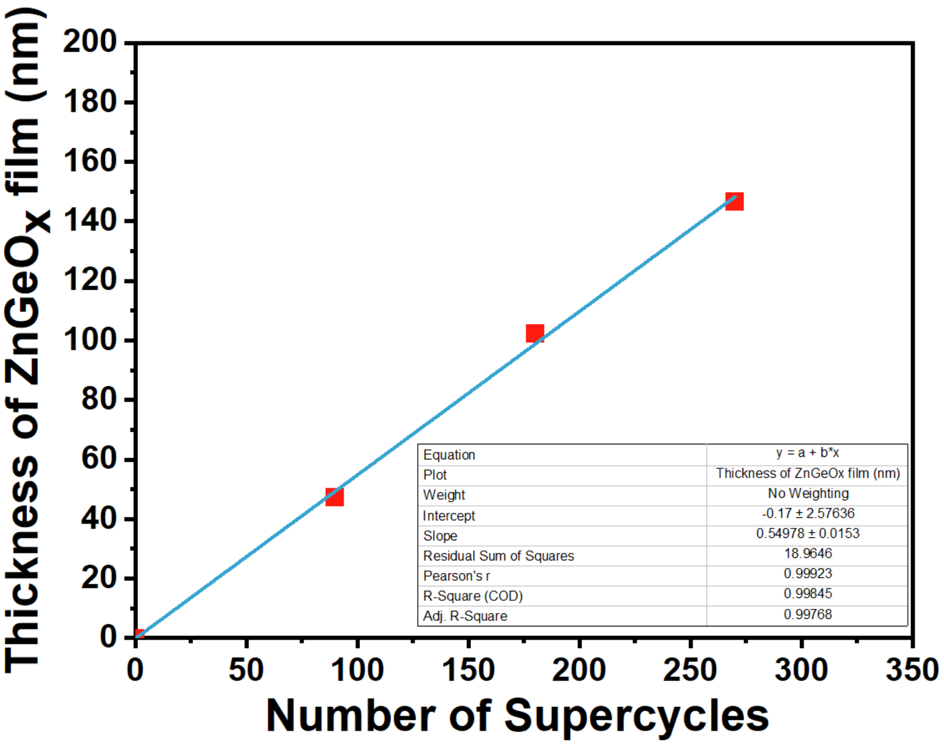

Supplementary Figure 4. ALD-$ZnGeO_x$ growth rate with linear fitting measured using a step profiler. The ZnGeOx films were deposited on clean Si substrates.

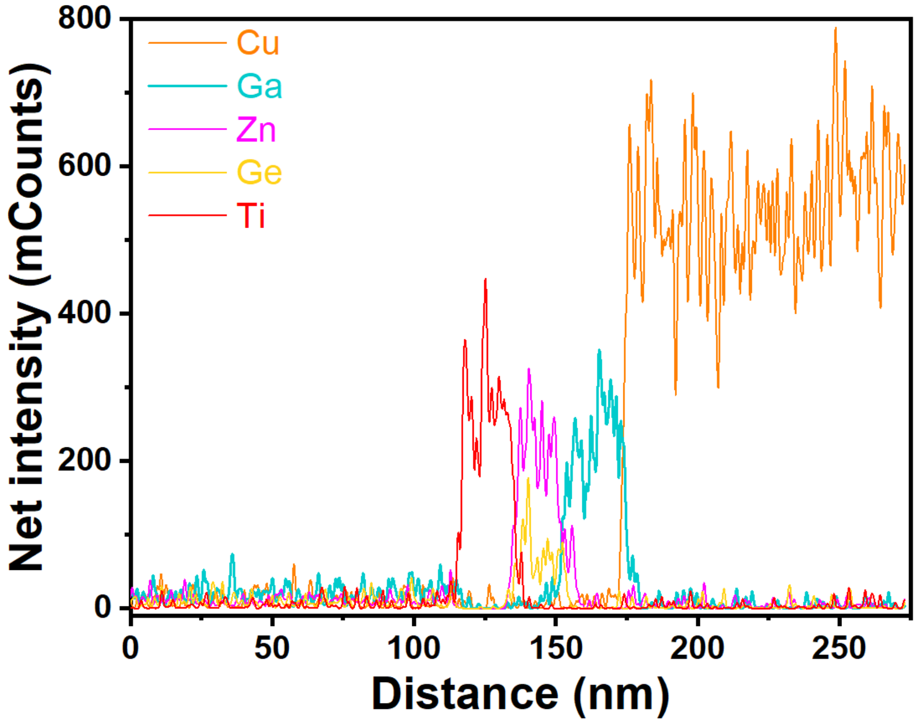

Supplementary Figure 5. The line profiles for Ti, Zn, Ge, Ga and Cu elements across the TiO_2, $ZnGeO_x$ and Ga_2O_3/Cu_2O interfaces.

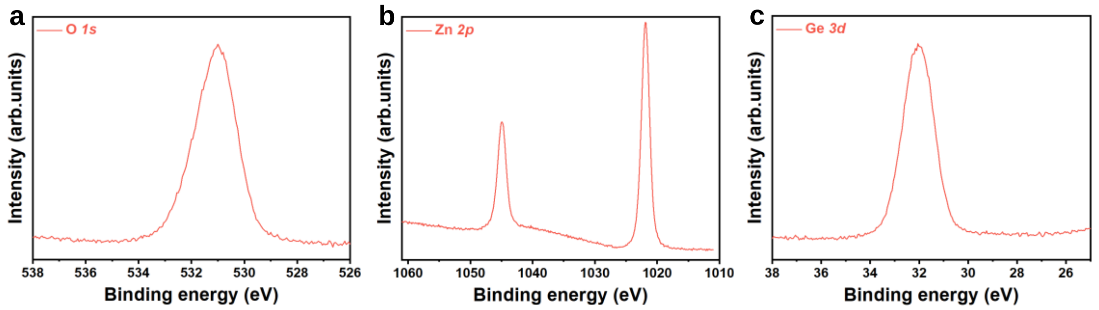

Supplementary Figure 6. Chemical composition of the 210 nm thick ALD-$ZnGeO_x$ film deposited on quartz substrate. a O *1s* core-level spectrum. **b** Zn *2p* core-level spectrum. **c** Ge *3d* core-level spectrum. The tables in the inset show the corresponding atomic content. The results of XPS tests show the atomic ratio of Zn, Ge and O is close to 2:1:4.

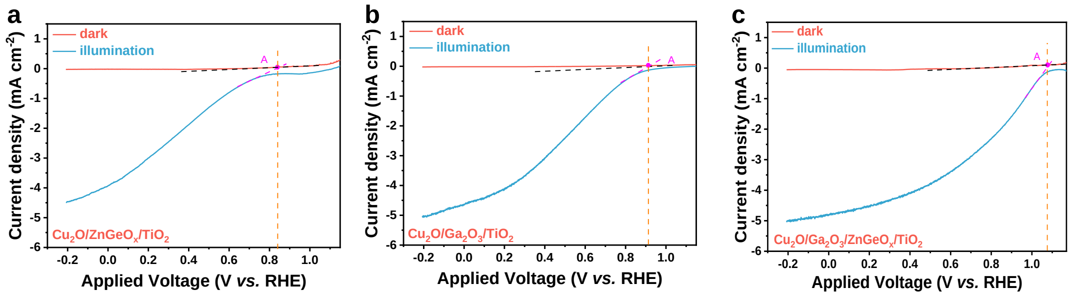

Supplementary Figure 7. Schematic diagram of the onset potential of **a**, the $Cu_2O/ZnGeO_x/TiO_2$ photocathode, **b**, the $Cu_2O/Ga_2O_3/TiO_2$ photocathode, and **c**, the $Cu_2O/Ga_2O_3/ZnGeO_x/TiO_2$ photocathode. Here, we define the onset potential as the potential value corresponding to the intercept (point A in the figure) between the extrapolated tangent lines of the *J*-*V* curve measured during illumination and in the dark.

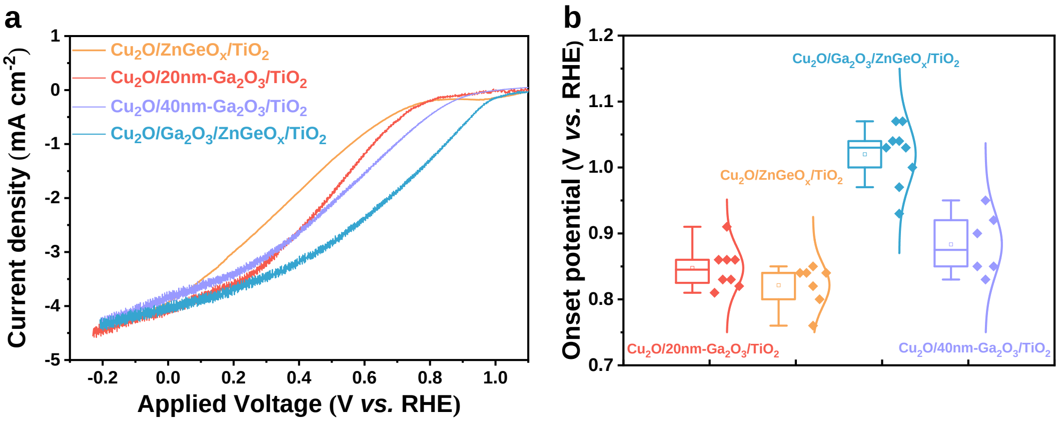

Supplementary Figure 8. Photoelectrochemical measurements. a *J*-*V* curves of different Cu_2O photocathodes under simulated AM 1.5G continuous illumination. **b** Statistical onset potential of variously prepared Cu_2O photocathodes.

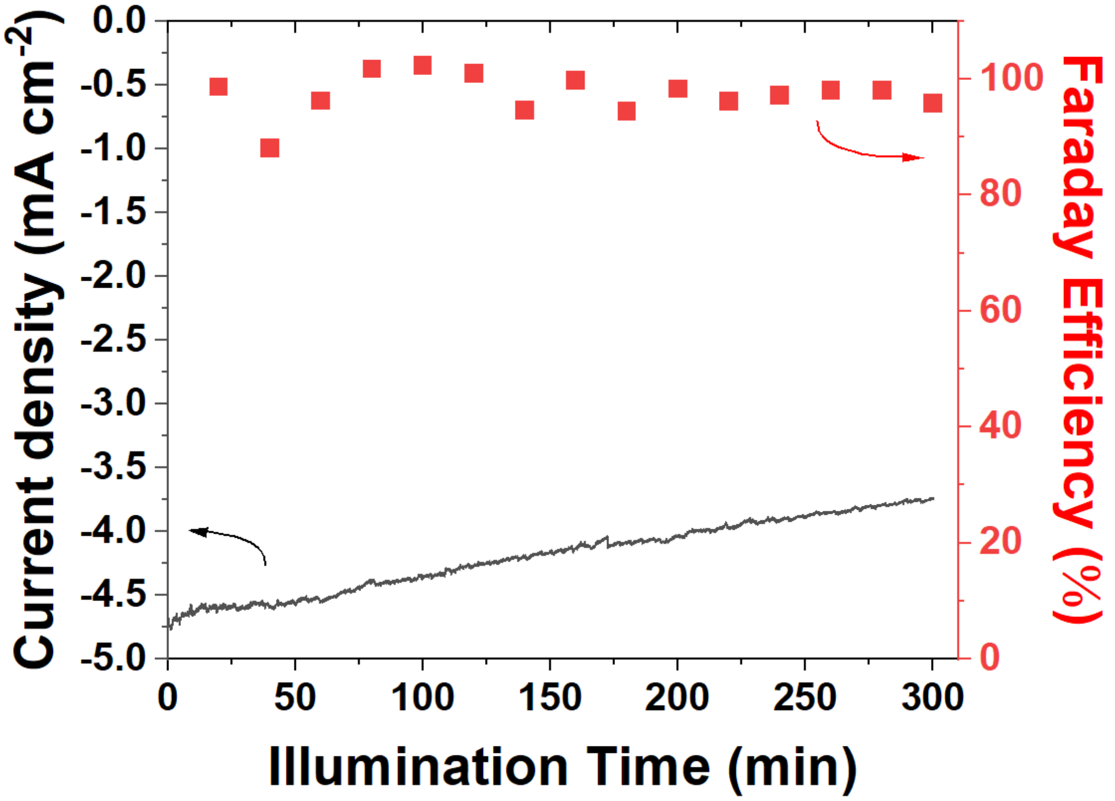

Supplementary Figure 9. The Faradaic efficiency test of the $Cu_2O/Ga_2O_3/ZnGeO_x/TiO_2$ photocathode for hydrogen evolution and corresponding real-time current density curve at 0 V_{RHE} under simulated AM 1.5G irradiation (100 mW cm^{-2}).

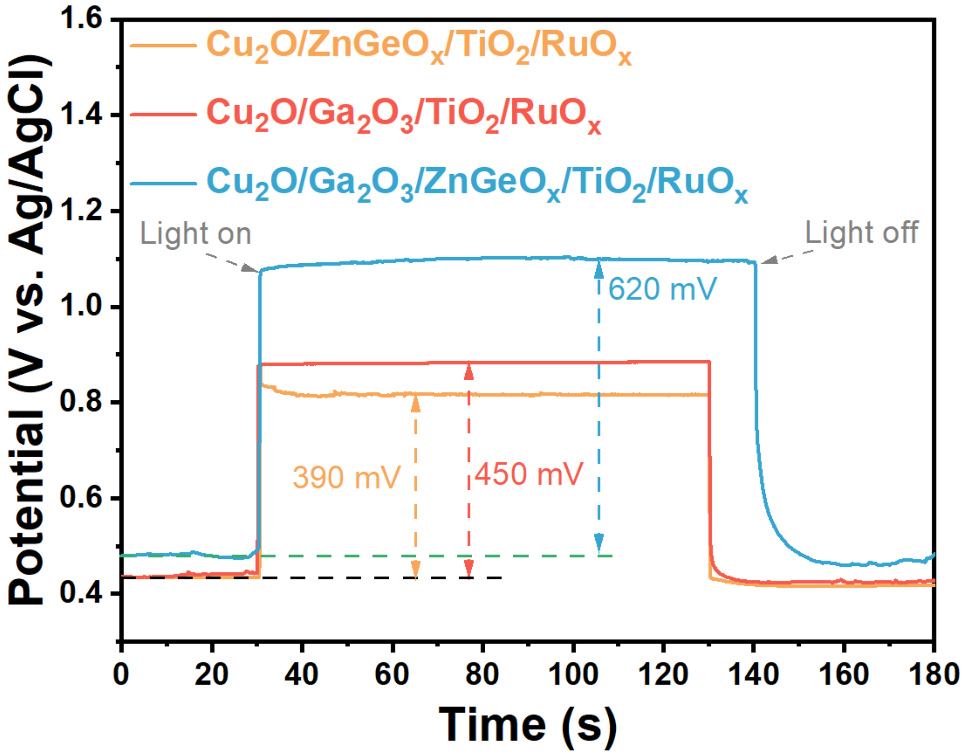

Supplementary Figure 10. The open-circuit potential of different Cu_2O photocathodes measured at chopped-light simulated AM 1.5G illumination (100 mW cm^{-2}) in a phosphate–sulfate buffer electrolyte (pH 5).

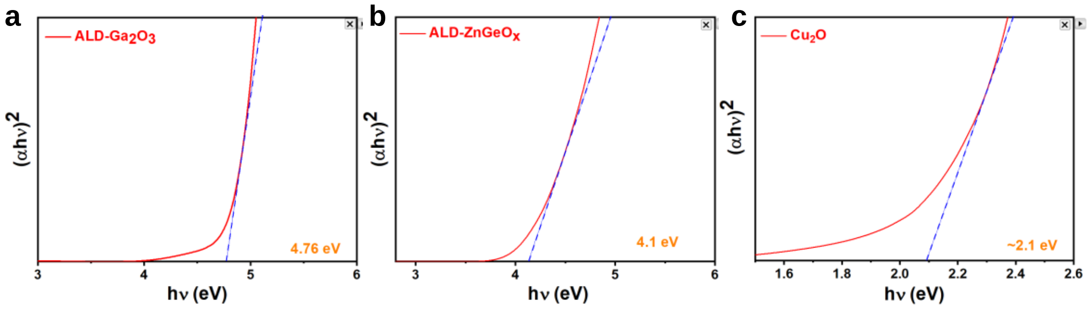

Supplementary Figure 11. Band gaps of different layers. a Tauc plot of ALD-Ga_2O_3 on quartz substrates with linear extrapolation. **b** Tauc plot of ALD-$ZnGeO_x$ on quartz substrates with linear extrapolation. **c** Tauc plot of Cu_2O on FTO substrate with linear extrapolation.

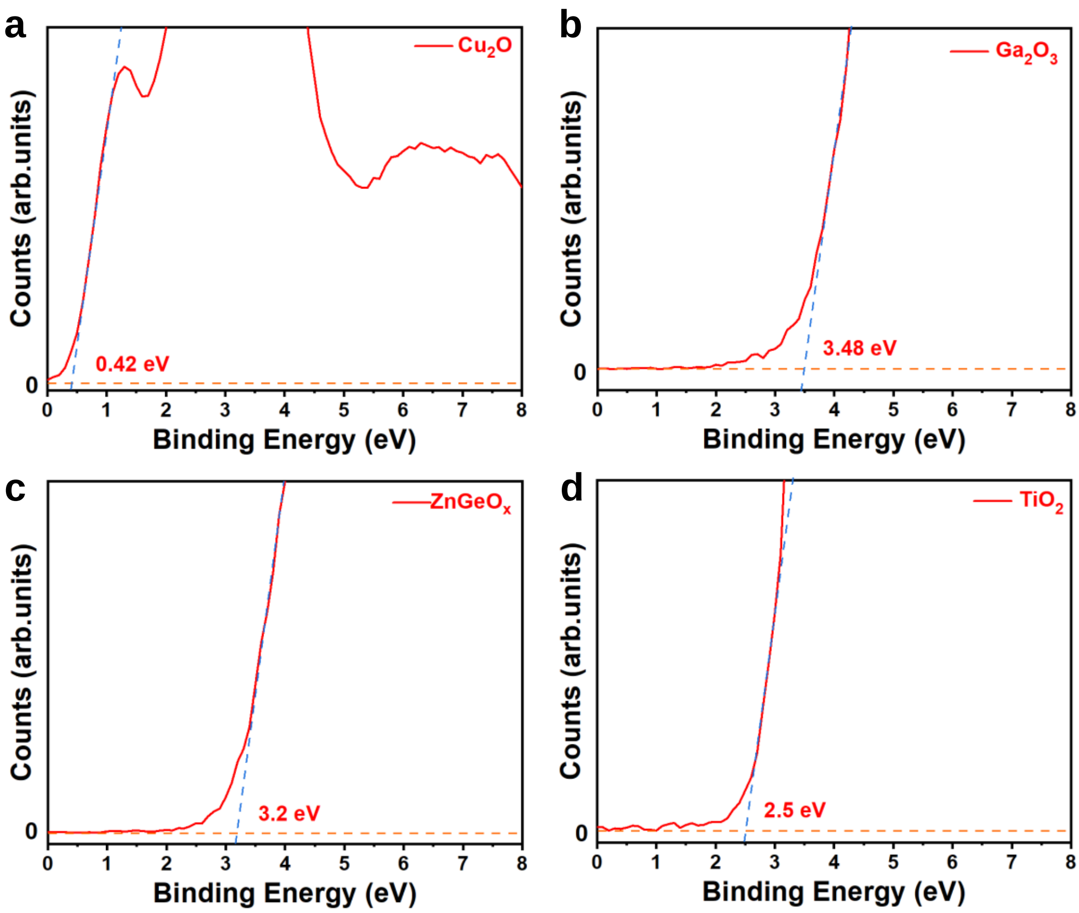

Supplementary Figure 12. The XPS valence band spectra. The XPS valence band spectra for the four different layers: **a**, Cu_2O, **b**, ALD-Ga_2O_3, **c**, ALD-$ZnGeO_x$ and **d**, ALD-TiO_2.

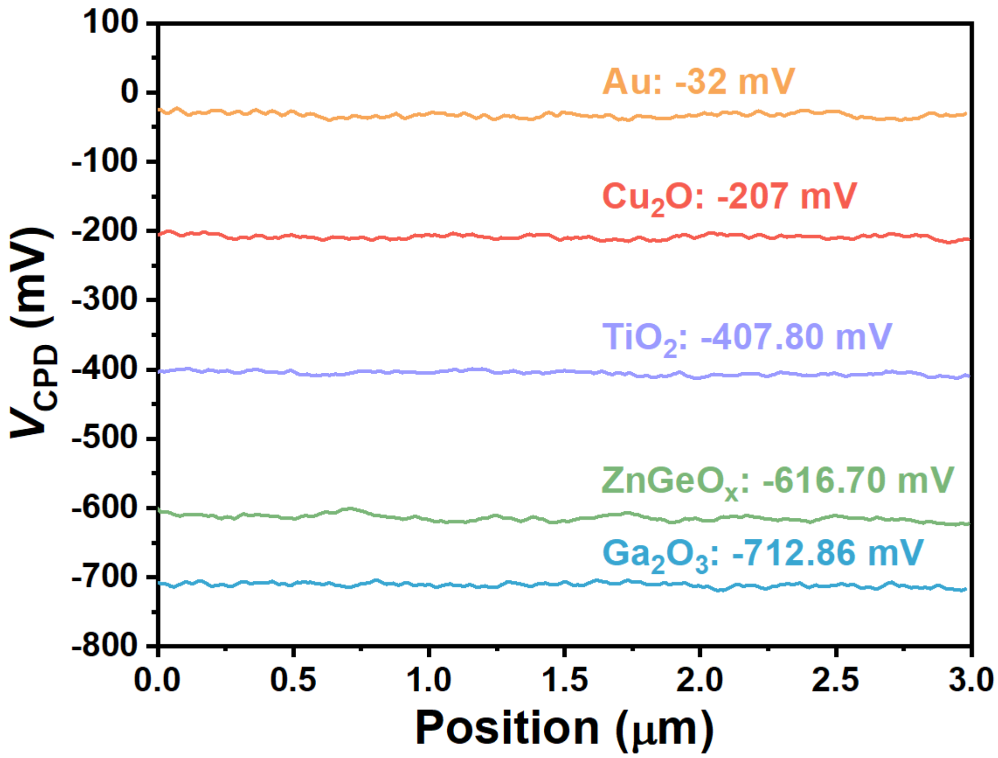

Supplementary Figure 13. The line distribution of the contact potential difference for the different samples, which are extracted from their KPFM images.

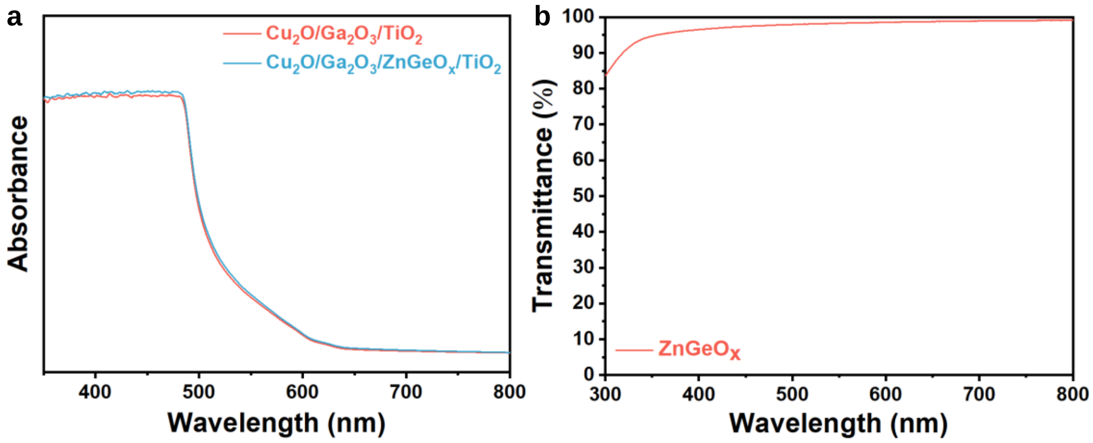

Supplementary Figure 14. Effect of $ZnGeO_x$ layer insertion on optical absorbance of the Cu_2O photocathode. **a** UV-vis absorption spectra of the $Cu_2O/Ga_2O_3/TiO_2$ photocathode and the $Cu_2O/Ga_2O_3/ZnGeO_x/TiO_2$ photocathode. The Cu_2O film was deposited on FTO substrate, and bare FTO glasses were used for baseline correction. **b** Transmittance spectrum of $ZnGeO_x$ (~20 nm) measured on quartz substrate. Before testing, quartz glasses were used for baseline correction.

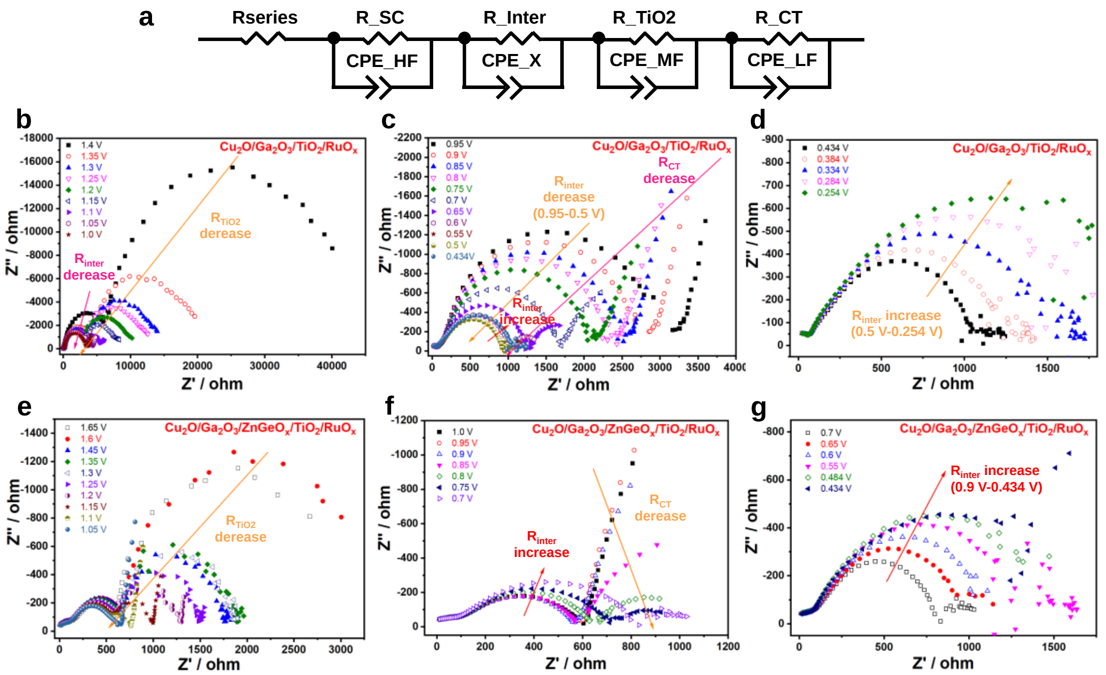

Supplementary Figure 15. EIS data. a The equivalent circuit model used to fit the EIS data of variously prepared Cu_2O-based photocathodes. **b-d** Nyquist plots of the $Cu_2O/Ga_2O_3/TiO_2/RuO_x$ photocathode and **e-g** the $Cu_2O/Ga_2O_3/ZnGeO_x/TiO_2/RuO_x$ photocathode at different bias potentials under illumination (light intensity of ~120 W m^{-2}).

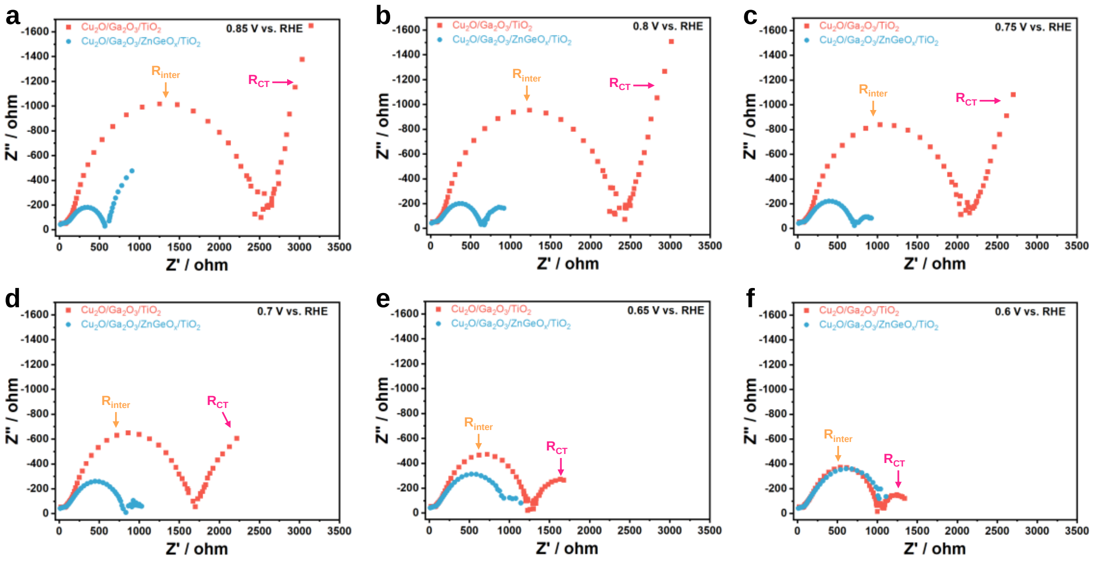

Supplementary Figure 16. EIS data. A direct comparison of the Nyquist plot the $Cu_2O/Ga_2O_3/TiO_2/RuO_x$ photocathode and the $Cu_2O/Ga_2O_3/ZnGeO_x/TiO_2/RuO_x$ photocathode at **a**, 0.85 V, **b**, 0.8 V, **c**, 0.75 V, **d**, 0.7 V, **e**, 0.65 V, and **f**, 0.85 V RHE under illumination (light intensity of ~120 W m^{-2}).

Supplementary Table 1. Frequency regions of the detected resistance under illumination.

Resistance	Frequency range
R_{SC}	100 kHz-10 kHz
R_{inter}	10 kHz-40 Hz
R_{TiO2}	1 kHz-mHz
R_{CT}	80 Hz-mHz

Supplementary Table 2. Selected representative results of reported single-junction Cu_2O photocathodes for PEC water splitting.

Device structure	Electrolyte (pH)	Saturated photocurrent density (mA cm^{-2})	Onset potential (V vs. RHE)	Ref.
Cu_2O/TiO_2	0.1 M sodium acetate (~6.5)	~0.70	~0.46	1
Cu_2O/1nm-TiO_2/PA/TiO_2/Pt	1 M phosphate solution (7)	4	0.50	2
Cu_2O/AZO/TiO_2/Pt	1 M Na_2SO_4 - 0.1 M phosphate solution (4.9)	7.6	0.50	3
Cu_2O/PUA/TiO_2/Pt	1 M Na_2SO_4 solution (7)	5.5	0.60	4
p-Cu_2O/n-Cu_2O/AZO/TiO_2/Pt	0.5 M Na_2SO_4 - 0.1 M KH_2PO_4 solution (4.15)	4.3	0.70	5
Cu_2O/ZnS/TiO_2/Pt	0.2 M KH_2PO_4 buffer solution (7)	~2.2	0.72	6
Cu_2O/UT-CuO/AZO/TiO_2/Pt	0.5 M Na_2SO_4 - 0.2 M potassium borate (5)	9	0.78	7
Cu_2O-NW/Ga_2O_3/TiO_2/RuO_x	0.5 M Na_2SO_4- 0.1 M KH_2PO_4 (5)	10	1.0	8
Cu_2O-NW/Ga_2O_3/TiO_2/Pt	0.5 M Na_2SO_4- 0.1 M KH_2PO_4 (4.26)	2.95	~1.02	9
Cu_2O/CTF-BTh/MoS_x	0.5 M Na_2SO_4 (6.8)	10.2	~1.0	10
Cu_2O/Ga_2O_3/$ZnGeO_x$/TiO_2/RuO_x	0.5 M Na_2SO_4- 0.1 M KH_2PO_4 (5)	5.1	1.07	This work

Supplementary References

1. Siripala, W., Ivanovskaya, A., Jaramillo, T. F., Baeck, S.-H. & McFarland, E. W. A Cu_2O/TiO_2 Heterojunction Thin Film Cathode for Photoelectrocatalysis. *Sol. Energy Mater. Sol. Cells* **77**, 229-237 (2003).
2. Wick-Joliat, R. et al. Stable and Tunable Phosphonic Acid Dipole Layer for Band Edge Engineering of Photoelectrochemical and Photovoltaic Heterojunction Devices. *Energy Environ. Sci.* **12**, 1901-1909 (2019).
3. Paracchino, A., Laporte, V., Sivula, K., Gratzel, M. & Thimsen, E. Highly Active Oxide Photocathode for Photoelectrochemical Water Reduction. *Nat. Mater.* **10**, 456-461 (2011).
4. Li, Y., Zhong, X. l., Luo, K. & Shao, Z. p. A Hydrophobic Polymer Stabilized p-Cu_2O Nanocrystal Photocathode for Highly Efficient Solar Water Splitting. *J. Mater. Chem. A* **7**, 15593-15598 (2019).
5. Wang, T. et al. Homogeneous Cu_2O p-n Junction Photocathodes for Solar Water Splitting. *Appl. Catal., B* **226**, 31-37 (2018).
6. Dai, P. d. et al. Forming Buried Junctions to Enhance the Photovoltage Generated by Cuprous Oxide in Aqueous Solutions. *Angew. Chem. Int. Ed.* **53**, 13493-13497 (2014).
7. Kim, D. S. et al. Atomically Tunable Photo-Assisted Electrochemical Oxidation Process Design for the Decoration of Ultimate-Thin CuO on Cu_2O Photocathodes and Their Enhanced Photoelectrochemical Performances. *J. Mater. Chem. A* **8**, 21744-21755 (2020).
8. Pan, L. f. et al. Boosting the Performance of Cu_2O Photocathodes for Unassisted Solar Water Splitting Devices. *Nat. Catal.* **1**, 412-420 (2018).
9. Li, C. l. et al. Positive Onset Potential and Stability of Cu_2O-Based Photocathodes in Water Splitting by Atomic Layer Deposition of a Ga_2O_3 Buffer Layer. *Energy Environ. Sci.* **8**, 1493-1500 (2015).
10. Zhang, Y., Lv, H. f., Zhang, Z., Wang, L., Wu, X. j. & Xu, H. x. Stable Unbiased Photo-Electrochemical Overall Water Splitting Exceeding 3% Efficiency via Covalent Triazine Framework/Metal Oxide Hybrid Photoelectrodes. *Adv. Mater.* **33**, 2008264 (2021).
