# Supplementary material for: Improving the photovoltage of Cu2O photocathodes with dual buffer layers
Source: Nat Commun. 2023 Nov 9;14:7228. doi: 10.1038/s41467-023-42799-x (PMC10636130; doi:10.1038/s41467-023-42799-x)
Supplement: Supplementary file 2 — Peer Review File [file 41467_2023_42799_MOESM2_ESM.pdf]

nature portfolio

Peer Review File

Improving the Photovoltage of Cu2O Photocathodes with Dual Buffer Layers

Open Access This file is licensed under a Creative Commons Attribution 4.0 International License, which permits use, sharing, adaptation, distribution and reproduction in any medium or format, as long as you give appropriate credit to the original author(s) and the source, provide a link to the Creative Commons license, and indicate if changes were made. In the cases where the authors are anonymous, such as is the case for the reports of anonymous peer reviewers, author attribution should be to 'Anonymous Referee' followed by a clear attribution to the source work. The images or other third party material in this file are included in the article's Creative Commons license, unless indicated otherwise in a credit line to the material. If material is not included in the article's Creative Commons license and your intended use is not permitted by statutory regulation or exceeds the permitted use, you will need to obtain permission directly from the copyright holder. To view a copy of this license, visit http://creativecommons.org/licenses/by/4.0/.

REVIEWER COMMENTS

Reviewer #1 (Remarks to the Author):

The manuscript by Cheng, Wu and Luo describes the optimization of the buffer layer/protection layer interface in Cu2O-based PEC electrodes, where a key resistance in the device is removed. The results are outstanding, and the proposed mechanism well justified by impedance spectroscopy and band alignment measurements. This is a very important development for Cu2O-based water splitting, and the strategy is indeed broadly applicable to other photoelectrode materials. The manuscript is very clearly written with very nice and clear figures, and the references are appropriate. I have only minor comments:

"under simulated AM 1.5G solar illumination" – please give power density as well. how was the light source power calibrated?

Fig. 4d,e: the absolute value of the R.inter resistance is notable as well, many times lower with the dual buffer layer

Reviewer #3 (Remarks to the Author):

The manuscript reported a Cu2O based photocathode with a high photovoltage of 1 V and a photocurrent of ~5 mA/cm2, using ZnGeOx buffer layer to eliminate the potential barrier at the interface of Ga2O3/TiO2. The typical Cu2O/Ga2O3/TiO2/cocatalyst structure with a considerable performance has been reported previously (for example, Niu et al, Catal. Sci. Technol., 2017, 7, 1602-1610), so this manuscript was lack of innovation even if the 0.15 V shift of onset potential and high onset of 1 V was achieved by the inserted ZnGeOx. Beside, in aspect of the details of the manuscript, some questions remain to be clarified.
1. Open-circuit potential should be provided and discussed from the perspective of photovoltage.
2. Why does the energy band bend downward from the interface to the TiO2 bulk when Ga2O3 or ZnGeOx contacts with TiO2 that has a lower Ef?
3. To make the EIS in Fig.S9 easier to interpret, the characteristic frequency of every resistance element should be provided or the corresponding fitting element of the EIS semicircles be marked. In addition, the kinetic overpotentials would also influence the onset potential. EIS results (Fig. 4d) show that Rct was decreased dramatically after ZnGeOx insertion at same bias, please explain and discuss its influence on the onset potential.
4. Supplement the UV-vis spectra and whether the inserted layer influences the optical absorbance.
5. In the experiment section, the thickness of Cu2O and TiO2 should also be given.
6. Please provide the performance comparison of the relevant Cu2O based photocathode reports.

Reviewer #4 (Remarks to the Author):

The manuscript titled "Improving the Photovoltage of Cu2O Photocathodes with Dual Buffer Layers" by Cheng and co-authors reported the addition of a buffer layer, ZnGeOx, into the commonly used Cu2O/Ga2O3/TiO2 photocathode structure. The use of buffer layer materials to increase the photovoltage of Cu2O photovoltaic devices has been extensively discussed in T. Minami's papers, such as ZnMgO (2012), AlGaO (2015), and ZnGeO (2016). The authors should cite these previous works to acknowledge the existing research. From this perspective, the novelty of this paper seems somewhat diminished. However, the concept of a "dual buffer layer" is interesting, especially since the authors demonstrated that the ZnGeOx buffer layer alone did not improve the photoelectrochemical (PEC) performance. With the dual buffer layer approach, the Cu2O photocathode exhibited a significantly

higher onset potential and a nice fill factor. Nevertheless, the study of the ZnGeOx layer in this paper appears insufficient, as it should be the central focus, and the explanation provided based on the Electrochemical Impedance Spectroscopy (EIS) data is not convincing. I will recommend accepting this manuscript after the following questions are addressed.

--The photocurrent at 0 V (RHE) is only 5 mA cm2, which is much lower than your previous work (around 6.5 mA/cm2 in Figure S10 in Pan, Nature Catalysis, 2018). Why?
--The definition of the onset potential is not mentioned in this paper, which could be controversial and may introduce a significant error range. Also, in Pan's paper, he claimed an onset potential over +1 V, while the author reports only 0.85 V in this manuscript with the same Ga2O3 buffer layer. The same standard should be used to compare these data.
--The term "ZnGeOx layer" is central to this manuscript and requires more accurate information. A clear stoichiometric ratio of Zn, Ge, O should be provided, along with the electrical properties and XRD analysis of the ZnGeOx layer.
--On line 150, calculating the thickness of the ZnGeOx film by measuring cross-sectional thickness is very inaccurate, especially on such a rough Cu2O surface. Such a statement is unscientific. Using a step profiler or ellipsometry would yield more precise and accurate results.
--On line 152, the step profiler should be shown in Figure S2.
In Figure 2c, the marking for different ALD layers appears arbitrary. It is challenging to observe a clear difference between them (at least in this figure). In contrast, the difference of ALD layers in Fig 2b is more apparent.
--In Figure 4b, the band bending of the ZnGeOx and TiO2 layers is misrepresented. For example, at the Ga2O3/TiO2 interface, since the Fermi level of TiO2 is lower than the H+/H2 energy level (some papers claim a higher Fermi level), the TiO2 should exhibit downward band bending.
--The fitting and extraction of Rct, Rsc, Rtio2, and Rinter from the EIS data should be clearly described in the supporting information.
--From Figure 4c, the most significant difference appears to be the Rct value; however, the authors attribute the improvement only to the Rinter value, which is not convincing. A direct comparison of the Nyquist plot at potentials between 0.6 and 0.8 V RHE will be more illustrative.
--The authors should provide a clearer explanation of the EIS data.
Please ensure that all the above-mentioned issues are addressed to enhance the manuscript's quality and credibility.

Point-by-point reply to reviewer comments

We thank all the reviewers for their valuable comments, which we have used to improve our work. Please find below the point-by-point reply to the comments, with the reply in blue color. The revisions made in the revised manuscript are highlighted in yellow color for easy inspection.

REVIEWER COMMENTS

Reviewer #1 (Remarks to the Author):

The manuscript by Cheng, Wu and Luo describes the optimization of the buffer layer/protection layer interface in Cu_2O-based PEC electrodes, where a key resistance in the device is removed. The results are outstanding, and the proposed mechanism well justified by impedance spectroscopy and band alignment measurements. This is a very important development for Cu_2O-based water splitting, and the strategy is indeed broadly applicable to other photoelectrode materials. The manuscript is very clearly written with very nice and clear figures, and the references are appropriate. I have only minor comments:

We thank the reviewer for the positive comments on our work.

Question 1

"under simulated AM 1.5G solar illumination" – please give power density as well. How was the light source power calibrated?

We thank the reviewer for the suggestion. The power density of the illumination source is 100 mW cm^{-2}, and it has been added to the revised manuscript. As for the calibration of light source power, a calibrated silicon reference diode with KG 3 filter was used to calibrate the light intensity of the illumination source. The short-circuit current of the calibrated silicon reference diode is fixed at 4.64 mA under simulated AM 1.5G solar illumination (100 mW cm^{-2}). Before each PEC performance test, the short-circuit current of the calibrated silicon reference diode reaches 4.64 mA by adjusting the optical path distance between the diode and the light source outlet, therefore ensuring that the light intensity is calibrated to 100 mW cm^{-2}. Finally, the sample is placed at the same position as the calibrated silicon reference diode for further PEC measurements.

Revised manuscript:

"For all the photocathodes, PEC measurements were performed in a phosphate–sulfate buffer electrolyte (pH 5) under simulated AM 1.5G solar illumination (100 mW cm^{-2}) in a standard three-electrode system."

Question 2

Fig. 4d, e: the absolute value of the $R_{.inter}$ resistance is notable as well, many times lower with the dual buffer layer.

We thank the reviewer for pointing this out. Indeed, the R_{inter} resistance is also lower with the dual buffer layer. We have added the following sentence in the revised manuscript.

Revised manuscript:
"Both of their R_{inter} values are almost constant before the H_2 evolution begins, and the R_{inter} of the $Cu_2O/Ga_2O_3/ZnGeO_x/TiO_2$ photocathode are smaller than that of the $Cu_2O/Ga_2O_3/TiO_2$ photocathode."

Reviewer #3 (Remarks to the Author):

The manuscript reported a Cu_2O based photocathode with a high photovoltage of 1 V and a photocurrent of ~5 mA/cm^2, using $ZnGeO_x$ buffer layer to eliminate the potential barrier at the interface of Ga_2O_3/TiO_2. The typical $Cu_2O/Ga_2O_3/TiO_2$/cocatalyst structure with a considerable performance has been reported previously (for example, Niu et al, Catal. Sci. Technol., 2017, 7, 1602-1610), so this manuscript was lack of innovation even if the 0.15 V shift of onset potential and high onset of 1 V was achieved by the inserted $ZnGeO_x$. Beside, in aspect of the details of the manuscript, some questions remain to be clarified.

We thank the reviewer for the comments, which help to improve the quality of our manuscript. The manuscript highlights the insertion of another buffer layer ($ZnGeO_x$) between the original buffer layer (Ga_2O_3) and the protection layer (TiO_2), which eliminates the unfavorable barrier at the Ga_2O_3/TiO_2 interface. As a result, the onset potential of the Cu_2O photocathode is shifted to 1.07 V (vs. RHE) rather than 1 V (vs. RHE). For the definition of the onset potential, previously, we define the potential when the photocurrent density increases under continuous illumination as the onset potential. It can be easily misinterpreted due to the influence of photocurrent increase magnitude. In the revised manuscript, we define the potential corresponding to the intercept between the extension tangent lines of the J-V curve under illumination and under dark respectively as the onset potential. For ease of understanding, we have also added the schematic diagram of the definition of the onset potential (Supplementary Figure 7) in the revised manuscript, where the potential corresponding to point A is the onset potential. Finally, the onset potential of the $Cu_2O/Ga_2O_3/ZnGeO_x/TiO_2$ photocathode was corrected to 1.07 V (RHE). The onset potentials of other photocathodes have also been corrected in the revised manuscript.

Moreover, for a clearer comparison, we took a screenshot of the J-V curves (Figure R1) from the reference literature (Niu et al, Catal. Sci. Technol., 2017, 7, 1602-1610) mentioned by the reviewer. We can see that the photocurrent density of the Cu_2O/Ga_2O_3/AZO/TiO_2/Pt photocathode does not tend to increase as the bias scans from 1 V to 0.9 V (vs. RHE). This part of photocurrent response is more likely due to the capacitance effect rather than the hydrogen evolution. In contrast, for the J-V curves of the $Cu_2O/Ga_2O_3/ZnGeO_x/TiO_2$ photocathode in our work (Figure 3), when the bias scans from 1.07 V to -0.2 V (vs. RHE), the photocurrent density increases gradually without any reverse decrease. Therefore, the onset potential in our results is better than the results in the literature mentioned by the reviewer. For the Cu_2O photocathode, we admit that Ga_2O_3 can form an optimal interfacial band alignment with Cu_2O, making the onset potential of the Cu_2O photocathode as high as 1 V (vs. RHE). Nevertheless, the onset potential in our results is 1.07 V, which is still a positive shift of 70 mV compared to the current state-of-the-art Cu_2O photocathodes (Pan et al, Nature Catalysis, 2018, 1, 412-420). Though it is a small step, it is an important advancement. Our work does not optimize the p-n junction of the Cu_2O interface, but reveals the effect of the unfavorable barrier between the buffer layer and the protective

layer on the onset potential. In addition, we propose to insert another buffer layer to eliminate this reverse effect, which provides a new strategy to improve the onset potential.

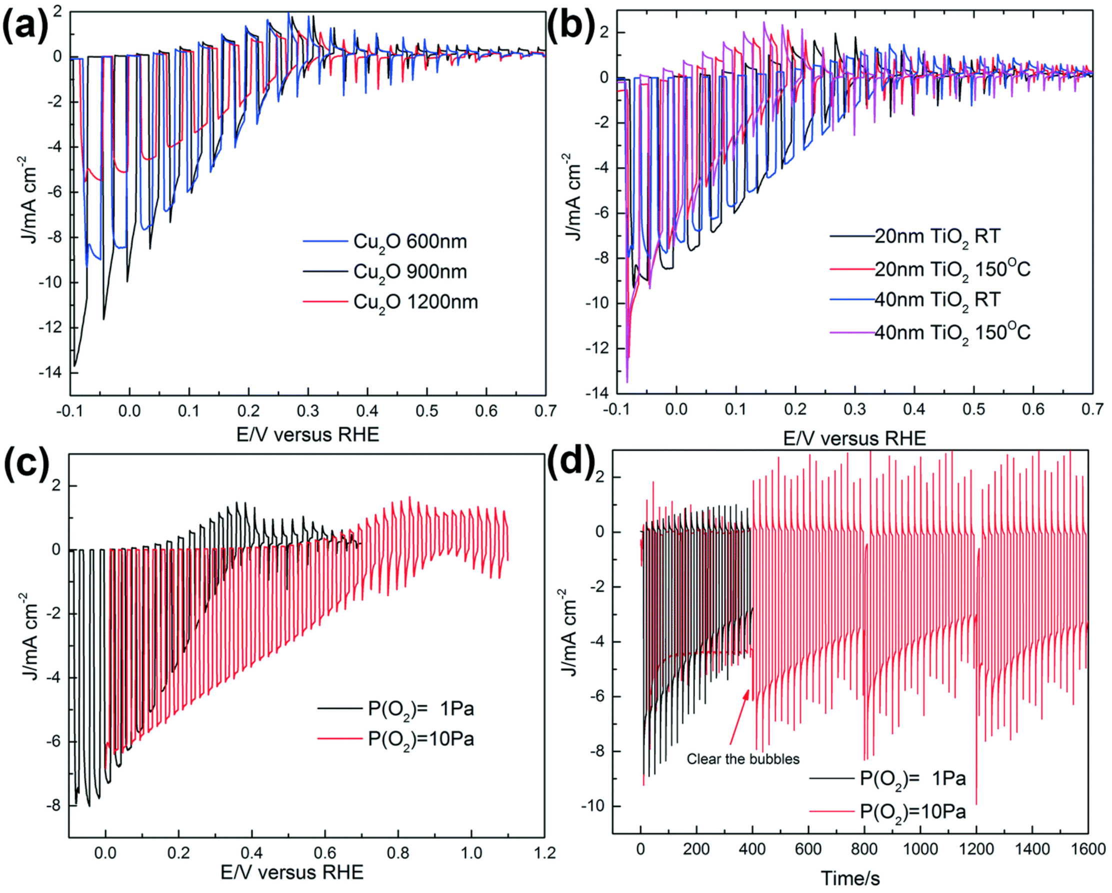

Fig. R1 PEC responses of electrodes with different thicknesses of Cu_2O (a), different thicknesses or different deposition temperatures of TiO_2 (b) and different oxygen pressures used for deposition of Ga_2O_3 (c). (d) Stability of the photocurrent at 0 V vs. RHE for the photocathodes in (c). Screenshots of these pictures are from the mentioned reference literature (Niu et al, Catal. Sci. Technol., 2017, 7, 1602-1610).

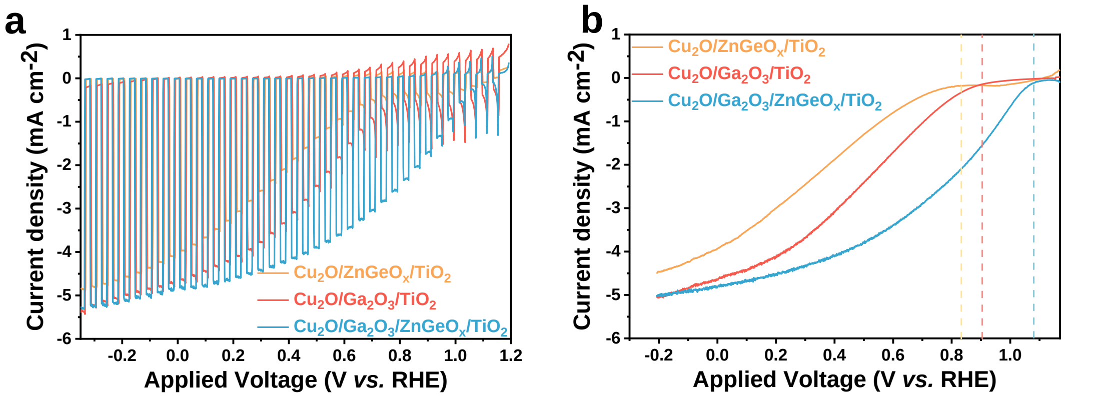

Figure 3. (a) J-V curves of different Cu_2O-based photocathodes under simulated AM 1.5G chopped illumination and (b) continuous illumination. The data are from our

manuscript.

Question 1

Open-circuit potential should be provided and discussed from the perspective of photovoltage.

We thank the reviewer for the suggestion. We have added the open-circuit potential curves of Cu_2O photocathodes under chopped-light illumination in the revised manuscript. The open-circuit potential curves are shown in Supplementary Figure 10 of the supporting information in the revised manuscript. The results show that the $Cu_2O/Ga_2O_3/ZnGeO_x/TiO_2/RuO_x$ photocathode has the largest open-circuit potential difference (the open-circuit potential under light subtracts the dark-state open-circuit potential). This phenomenon implies that the $Cu_2O/Ga_2O_3/ZnGeO_x/TiO_2/RuO_x$ photocathode possesses an enhanced carrier separation efficiency and an increased photovoltage, and the corresponding discussion was added to the revised manuscript.

Revised manuscript:

"Moreover, the open-circuit potential curves (Figure S10) show that the open-circuit potential difference of the $Cu_2O/Ga_2O_3/ZnGeO_x/TiO_2/RuO_x$ photocathode (620 mV) is larger than that of the $Cu_2O/Ga_2O_3/TiO_2/RuO_x$ photocathode (450 mV) and the $Cu_2O/ZnGeO_x/TiO_2/RuO_x$ photocathode (390 mV), which further suggests that the $Cu_2O/Ga_2O_3/ZnGeO_x/TiO_2/RuO_x$ photocathode possesses an enhanced carrier separation efficiency and an increased photovoltage."

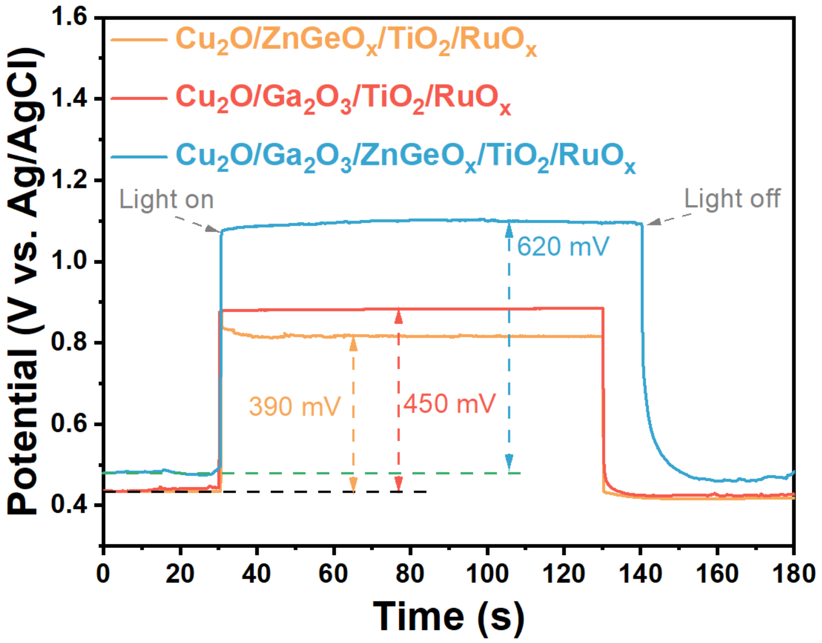

Supplementary Figure 10. The open-circuit potential of different Cu_2O photocathodes measured at chopped-light simulated AM 1.5G illumination (100 mW cm^{-2}) in a phosphate–sulfate buffer electrolyte (pH 5).

Question 2

Why does the energy band bend downward from the interface to the TiO_2 bulk when Ga_2O_3 or $ZnGeO_x$ contacts with TiO_2 that has a lower E_f?

We thank the reviewer for pointing out this, which helps to improve the quality of our manuscript. The reviewer is correct. We made a mistake on the band bending of Ga_2O_3 or $ZnGeO_x$ when contacts with TiO_2. In fact, the energy band bending should be upward from the interface to the TiO_2 bulk when Ga_2O_3 or $ZnGeO_x$ contacts with TiO_2. We have corrected this error in the revised manuscript.

Revised manuscript:

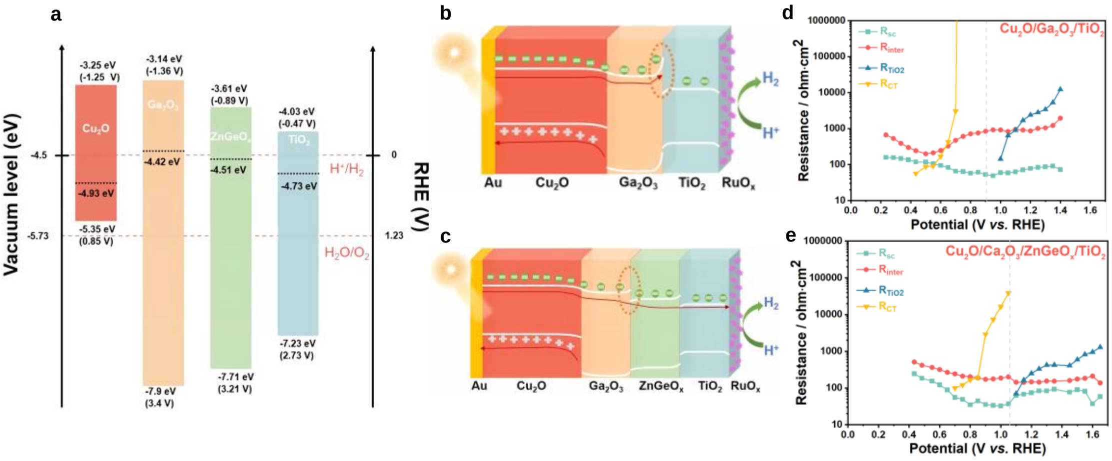

Figure 4. (a) Energy diagrams of Cu_2O, Ga_2O_3, $ZnGeO_x$, and TiO_2 (from left to right) derived from Kelvin probe force microscopy (KPFM), XPS valence band spectra and the Tauc plots. (b) Schematic diagram of the photogenerated carrier transport behavior in the $Cu_2O/Ga_2O_3/TiO_2/RuO_x$ photocathode and (c) the $Cu_2O/Ga_2O_3/ZnGeO_x/TiO_2/RuO_x$ photocathode under illumination, respectively. (d) Resistances from the EIS fitting of the $Cu_2O/Ga_2O_3/TiO_2/RuO_x$ photocathode and (e) the $Cu_2O/Ga_2O_3/ZnGeO_x/TiO_2/RuO_x$ photocathode under illumination, respectively.

Question 3

To make the EIS in Fig.S9 easier to interpret, the characteristic frequency of every resistance element should be provided or the corresponding fitting element of the EIS semicircles be marked. In addition, the kinetic overpotentials would also influence the onset potential. EIS results (Fig. 4d) show that R_{ct} was decreased dramatically after $ZnGeO_x$ insertion at same bias, please explain and discuss its influence on the onset potential.

We thank the reviewer for the suggestion. We have added the characteristic frequency of every resistance element in the revised manuscript. The specific parameters are presented in Supplementary Table 1 of the supporting information. In addition, the

corresponding fitting resistance elements of the EIS semicircles were also marked in the Supplementary Figure 15 for easy understanding.

The RuO_x is used as the co-catalyst for HER in both the $Cu_2O/Ga_2O_3/TiO_2$ photocathode and the $Cu_2O/Ga_2O_3/ZnGeO_x/TiO_2$ photocathode, and the $ZnGeO_x$ buffer layer is not in direct contact with RuO_x. Therefore, the kinetic overpotential is identical for the two photocathodes. Combined with the open-circuit potential test results (Supplementary Figure 10), we can see that the insertion of $ZnGeO_x$ mainly increases the photovoltage. In addition, the low frequency resistance, R_{CT}, represents the hydrogen-generating charge transfer over the hydrogen evolution catalyst into the electrolyte solution. However, before hydrogen evolution, the RuO_x catalyst needs to be reduced to RuO_x^{-1}, that is, Ru(IV) needs to be reduced to Ru(III). R_{CT} is related to the current flow for the reduction step of RuO_x. Once the bias exceeds the onset potential of HER, as the bias becomes more negative, more electrons will flow through the RuO_x catalyst to reach the electrolyte, accelerating the reduction of RuO_x and sharply reducing the R_{CT}. We think that after the insertion of $ZnGeO_x$, the construction of energy level gradient increases the photovoltage and facilitates photogenerated electrons to the catalyst, which accelerates the reduction of RuO_x. Therefore, it is reasonable that R_{CT} of the $Cu_2O/Ga_2O_3/ZnGeO_x/TiO_2$ photocathode is smaller than that of the $Cu_2O/Ga_2O_3/TiO_2$ photocathode at the same bias. In other words, the adverse barrier at the Ga_2O_3/TiO_2 interface is unfavorable to photogenerated electrons migration, resulting in slow RuO_x reduction and late onset potential.

Revised manuscript:

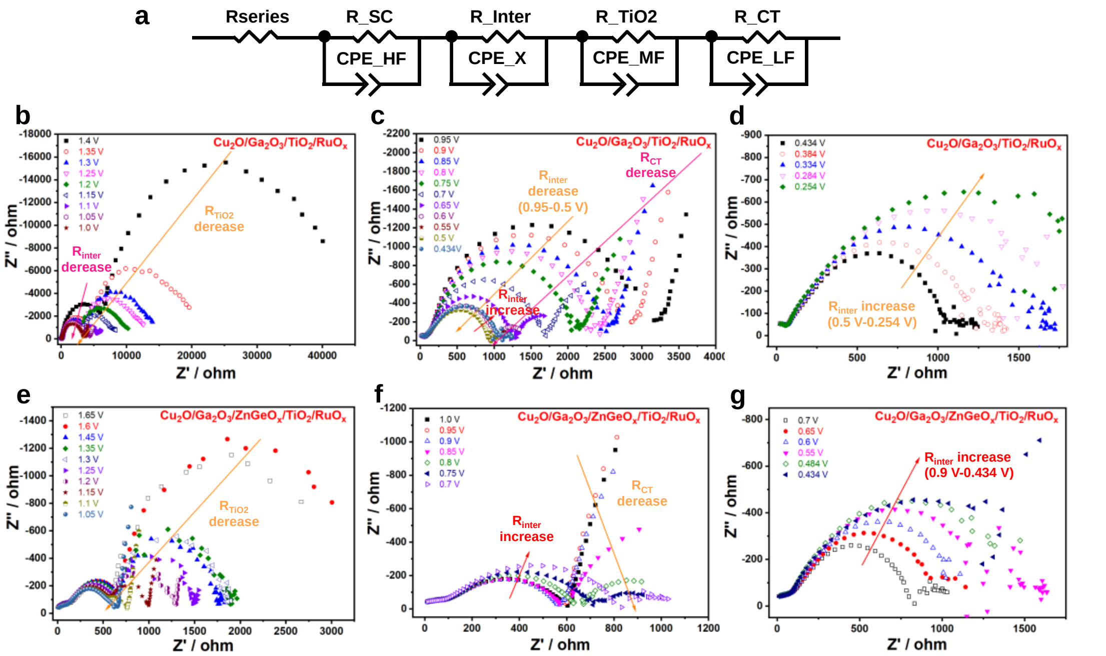

Supplementary Figure 9. (a) The equivalent circuit model used to fit the EIS data of variously prepared Cu_2O-based photocathodes. (b-d) Nyquist plots of the $Cu_2O/Ga_2O_3/TiO_2/RuO_x$ photocathode and (e-g) the $Cu_2O/Ga_2O_3/ZnGeO_x/TiO_2/RuO_x$ photocathode at different bias potentials under illumination (light intensity of ~120 W

m^{-2}).

Supplementary Table 1. Frequency regions of the detected resistance under illumination.

Resistance	Frequency range
R_{SC}	100 kHz-10 kHz
R_{inter}	10 kHz-40 Hz
R_{TiO2}	1 kHz-mHz
R_{CT}	80 Hz-mHz

Question 4

Supplement the UV-vis spectra and whether the inserted layer influences the optical absorbance.

We thank the reviewer for the suggestion. The UV-vis spectra have been supplemented in Supplementary Figure 14a in the revised manuscript. From the results of the UV-vis spectra, it can be seen there is no significant change in optical absorbance after the insertion of $ZnGeO_x$. In fact, from the transmittance spectrum of $ZnGeO_x$ (Supplementary Figure 14b), we can also see that ZnGeOx has a low absorbance and a large transmittance in the range of 400-800 nm. Therefore, it is reasonable that there is no significant change in optical absorbance after insertion of $ZnGeO_x$.

Revised manuscript:

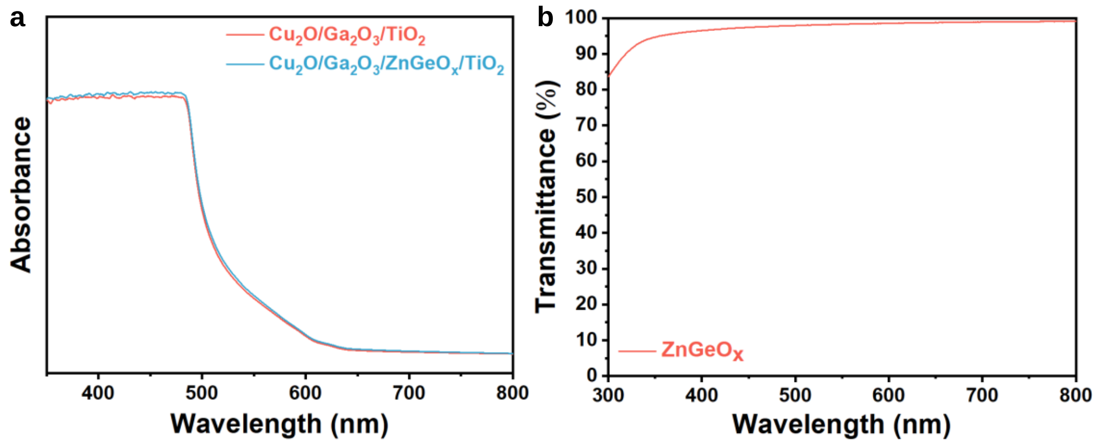

Supplementary Figure 14. (a) UV-vis absorption spectra of the $Cu_2O/Ga_2O_3/TiO_2$ photocathode and the $Cu_2O/Ga_2O_3/ZnGeO_x/TiO_2$ photocathode. The Cu_2O film was deposited on FTO substrate, and bare FTO glasses were used for baseline correction. (b) Transmittance spectrum of $ZnGeO_x$ (~20 nm) measured on quartz substrate. Before testing, quartz glasses were used for baseline correction.

Question 5

In the experiment section, the thickness of Cu_2O and TiO_2 should also be given.

We thank the reviewer for the suggestion. We have added the corresponding data of the thickness of Cu_2O and TiO_2 to the revised manuscript.

Revised manuscript:

"The electrodeposition time of all Cu_2O films is 100 min, which results in a thickness of Cu_2O film of about 860 nm."

"For scanning voltammetry testing and the measurement of open-circuit potential, the thickness of the TiO_2 protective layer is 20 nm, while for stability testing and the Faraday efficiency measurements, the thickness of TiO_2 protective layer is 120 nm."

Question 6

Please provide the performance comparison of the relevant Cu_2O based photocathode reports.

We thank the reviewer for the suggestion. The performance comparison of the relevant Cu_2O-based photocathode reports has been supplemented in Supplementary Table 2 of the supporting information in the revised manuscript.

Revised manuscript:

Supplementary Table 2. Selected representative results of reported single-junction Cu2O photocathodes for PEC water splitting.

Device structure	Electrolyte (pH)	Saturated photocurrent density (mA cm^{-2})	Onset potential (V vs. RHE)	Ref.
Cu_2O/TiO_2	0.1 M sodium acetate (~6.5)	~0.70	~0.46	4
Cu_2O/1nm-TiO_2/PA/TiO_2/Pt	1 M phosphate solution (7)	4	0.50	5
Cu_2O/AZO/TiO_2/Pt	1 M Na_2SO_4 - 0.1 M phosphate solution (4.9)	7.6	0.50	6
Cu_2O/PUA/TiO_2/Pt	1 M Na_2SO_4 solution (7)	5.5	0.60	7

p-Cu_2O/n-Cu_2O/AZO/TiO_2/Pt	0.5 M Na_2SO_4 - 0.1 M KH_2PO_4 solution (4.15)	4.3	0.70	8
Cu_2O/ZnS/TiO_2/Pt	0.2 M KH_2PO_4 buffer solution (7)	~2.2	0.72	9
Cu_2O/UT-CuO/AZO/TiO_2/Pt	0.5 M Na_2SO_4 -0.2 M potassium borate (5)	9	0.78	10
Cu_2O-NW/Ga_2O_3/TiO_2/RuO_x	0.5 M Na_2SO_4-0.1 M KH_2PO_4 (5)	10	~1.0	11
Cu_2O-NW/Ga_2O_3/TiO_2/Pt	0.5 M Na_2SO_4-0.1 M KH_2PO_4 (4.26)	2.95	~1.0	12
Cu_2O/CTF-BTh/MoS_x	0.5 M Na_2SO_4 (6.8)	10.2	~1.0	13
Cu_2O/Ga_2O_3/$ZnGeO_x$/TiO_2/RuO_x	0.5 M Na_2SO_4-0.1 M KH_2PO_4 (5)	5.1	1.07	This work

Reviewer #4 (Remarks to the Author):
The manuscript titled "Improving the Photovoltage of Cu_2O Photocathodes with Dual Buffer Layers" by Cheng and co-authors reported the addition of a buffer layer, $ZnGeO_x$, into the commonly used $Cu_2O/Ga_2O_3/TiO_2$ photocathode structure. The use of buffer layer materials to increase the photovoltage of Cu_2O photovoltaic devices has been extensively discussed in T. Minami's papers, such as ZnMgO (2012), AlGaO (2015), and ZnGeO (2016). The authors should cite these previous works to acknowledge the existing research. From this perspective, the novelty of this paper seems somewhat diminished. However, the concept of a "dual buffer layer" is interesting, especially since the authors demonstrated that the ZnGeOx buffer layer alone did not improve the photoelectrochemical (PEC) performance. With the dual buffer layer approach, the Cu_2O photocathode exhibited a significantly higher onset potential and a nice fill factor. Nevertheless, the study of the ZnGeOx layer in this paper appears insufficient, as it should be the central focus, and the explanation provided based on the Electrochemical Impedance Spectroscopy (EIS) data is not convincing. I will recommend accepting this manuscript after the following questions are addressed.

We thank the reviewer for the positive comments, which help to improve the quality of our manuscript. There is no doubt that Minami et al. have made an important contribution to improving the photovoltage of Cu_2O-based solar cells by developing various interface layers to optimize the p-n junction between Cu_2O and the n-type buffer layer. As suggested by the reviewer, the previous works of Minami's group have been cited in the revised manuscript. Compared to previous reports, our work reveals the importance of optimizing the band alignment between the n-type buffer layer and the protective layer, rather than focusing on optimizing the p-n junction interface, which makes the manuscript an innovative work.

Revised manuscript:
"Here, it is worth noting that Minami et al. focused on the development of new buffer layers (such as ZnMgO, AlGaO and ZnGeO) and the optimization of p-n junction, which made an important contribution to improving the photovoltage of Cu_2O solar cell.[39-41] In addition, they also emphasized the importance of buffer layer preparation methods.[42]"

Question 1
The photocurrent at 0 V (RHE) is only 5 mA cm^2, which is much lower than your previous work (around 6.5 mA/cm^2 in Figure S10 in Pan, Nature Catalysis, 2018). Why?

We thank the reviewer for checking our work in detail and pointing out this. The reviewer is correct. The photocurrent density in this work is lower than that of our previous work. The previous work (Pan et al, Nature Catalysis, 2018, 1, 412-420) was done in the laboratory of Professor Michael Grätzel at the École Polytechnique Fédérale de Lausanne in Switzerland. This work was done independently in our newly established laboratory at Nankai University in China. For the Cu_2O-based device,

especially since it contains many layers, the preparation parameters of each layer and the interface contact between every layer will greatly affect the performance of the resulting device. Due to the difference of chemicals and equipment, the preparation parameters of each layer need to be finely tuned and optimized. For example, an intuitive example is that the number of deposition cycles required for the current deposition of 20 nm Ga_2O_3 is different from the previous parameters. In addition, in Professor Michael Grätzel's laboratory, the preparation and optimization of the Cu_2O photocathode have been explored by many experienced scientific researchers for more than a decade. However, the first author of this work is a new student to the Cu_2O photocathode field, and has limited experience. With further efforts from several students, we believe we can get higher photocurrent density in the future.

Question 2

The definition of the onset potential is not mentioned in this paper, which could be controversial and may introduce a significant error range. Also, in Pan's paper, he claimed an onset potential over +1 V, while the author reports only 0.85 V in this manuscript with the same Ga_2O_3 buffer layer. The same standard should be used to compare these data.

We thank the reviewer for the suggestion, which helps to improve the quality of our manuscript. For the results in Pan's paper, we admit that the onset potential of the $Cu_2O/Ga_2O_3/TiO_2/RuO_x$ photocathode can reach 1 V (vs. RHE). As for why the onset potential of our photocathode with the same structure is only about 0.85 V, we speculate that the band alignment at the Cu_2O/Ga_2O_3 interface for our sample is not optimal. The interfacial defects of Cu_2O are a possible reason. In addition, the deposition parameters of Ga_2O_3 and TiO_2 overlayers can also affect the photovoltage of Cu_2O device (Niu et al, Catal. Sci. Technol., 2017, 7, 1602-1610; Li et al, Energy Environ. Sci., 2015, 8, 1493-1500). Moreover, some reports also obtained the onset potential of 0.85-0.9 V (vs. RHE) (such as Adv. Energy Mater. 2017, 1702323 and Energy Environ. Sci., 2022,15, 2002-2010) for the Cu_2O photocathode with the $Cu_2O/Ga_2O_3/TiO_2$ structure. The performance parameters are closely related to the preparation details.

For the definition of the onset potential, previously, we define the potential when the photocurrent density increases under continuous illumination as the onset potential. In the revised manuscript, we define the potential corresponding to the intercept between the extension tangent lines of the J-V curve under illumination and under dark respectively as the onset potential. For ease of understanding, we have also added the schematic diagram of the definition of the onset potential (Supplementary Figure 7) in the revised manuscript, where the potential corresponding to point A is the onset potential. Finally, the onset potential of the $Cu_2O/ZnGeO_x/TiO_2$ photocathode was corrected to 1.07 V (RHE). The onset potential of other photocathodes has also been corrected in the revised manuscript.

Revised manuscript:

"Here, we define the potential corresponding to the intercept between the extension tangent lines of the J-V curves under illumination (AM 1.5 G, 100 mW cm^{-2}) and under dark respectively as the onset potential (Supplementary Figure 7). In addition, when the bias becomes more negative, the photocurrent density should show a trend of continuous increase. As shown in Figure 3b, the onset potential of the $Cu_2O/Ga_2O_3/ZnGeO_x/TiO_2$ photocathode is 1.07 V (RHE), and has a 0.16 V (RHE) positive shift relative to that of the $Cu_2O/Ga2O_3/TiO_2$ photocathode (0.91 V vs. RHE)."

"From the results depicted in Figure 3a-c, the $Cu_2O/ZnGeO_x/TiO_2$ photocathode shows a lower saturated photocurrent density (~4.7 mA cm^{-2}), a later onset potential (0.84 V vs. RHE) and an unsatisfactory fill factor."

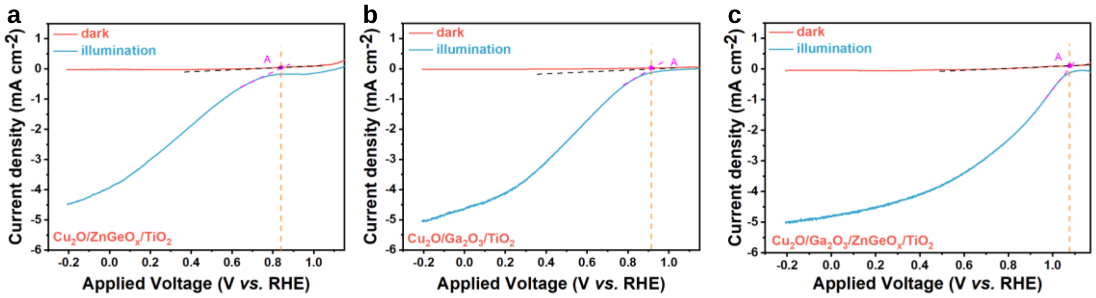

Supplementary Figure 7. Schematic diagram of the onset potential of **a**, the $Cu_2O/ZnGeO_x/TiO_2$ photocathode, **b**, the $Cu_2O/Ga_2O_3/TiO_2$ photocathode, and **c**, the $Cu_2O/Ga_2O_3/ZnGeO_x/TiO_2$ photocathode. Here, we define the potential corresponding to the intercept (point A in the figure) between the extension tangent lines of the J-V curve under illumination and under dark respectively as the onset potential.

Question 3

The term "$ZnGeO_x$ layer" is central to this manuscript and requires more accurate information. A clear stoichiometric ratio of Zn, Ge, O should be provided, along with the electrical properties and XRD analysis of the ZnGeOx layer.

We thank the reviewer for the suggestion, which helps to improve the quality of our manuscript. For the $ZnGeO_x$ layer, we conducted the XPS tests to analyze its chemical composition (Supplementary Figure 6). As a result, the atomic ratio of Zn, Ge and O is about 21.57:10.64:44.46, which is close to 2:1:4. Therefore, the approximate chemical formula of the $ZnGeO_x$ is $Zn_2Ge_1O_4$. We also supplemented the XRD pattern of $ZnGeO_x$, as shown in Supplementary Figure 1 in the revised manuscript. The $ZnGeO_x$ films deposited on quartz substrate and FTO substrate have no additional diffraction peaks except diffraction peaks from quartz substrate and FTO. This result shows that the $ZnGeO_x$ films are amorphous.

As for the electrical measurements of $ZnGeO_x$, we tested its resistance through the four-probe resistance test mode. Unfortunately, the resistance of $ZnGeO_x$ is too large to measure accurately. When the $ZnGeO_x$ film (~210 nm) was deposited on FTO

substrate, a resistance of 180 kΩ m^2 was obtained. However, when the $ZnGeO_x$ film (~210 nm) was deposited on quartz substrate, we failed to measure its resistance. Ideally, the film should be deposited on an insulating substrate for square resistance and Hall tests. Therefore, we are sorry that we cannot provide accurate electrical parameters such as square resistance and carrier mobility. This is also consistent with Minami *et al.*'s findings that the resistivity of the $Zn_{0.38}Ge_{0.62}$-O thin films was difficult to measure because of the difficulty of forming ohmic contacts on the amorphous films (Minami et al., Appl. Phys. Express, 2016, 9, 052301).

Revised manuscript:

"We conducted the X-ray photoelectron spectroscopy tests to analyze the chemical composition of $ZnGeO_x$ films (Supplementary Fig. 6), as a result, the atomic ratio of Zn, Ge and O is about 21.57:10.64:44.46, which is close to 2:1:4. Therefore, the approximate chemical formula of the $ZnGeO_x$ is Zn_2GeO_4. Therefore, all references of $ZnGeO_x$ below refer to Zn_2GeO_4."

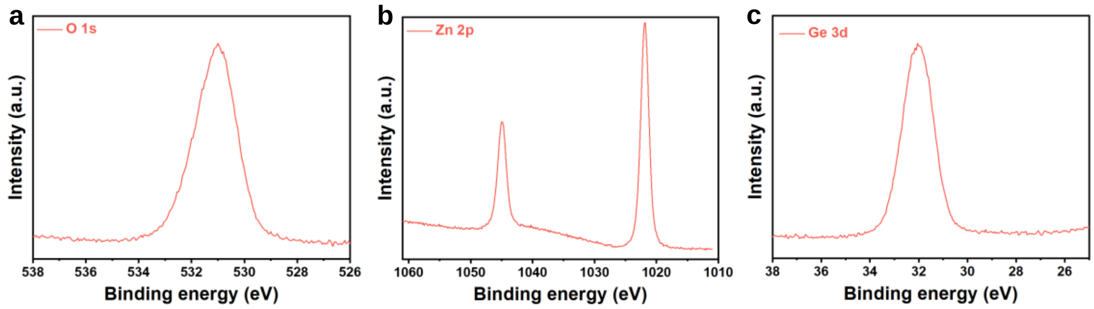

Supplementary Figure 6. Chemical composition of the 210 nm thick ALD-$ZnGeO_x$ film deposited on quartz substrate. (a) O 1s core-level spectrum. (b) Zn 2p core-level spectrum. (c) Ge 3d core-level spectrum. The tables in the inset show the corresponding atomic content. The results of XPS tests show the atomic ratio of Zn, Ge and O is close to 2:1:4.

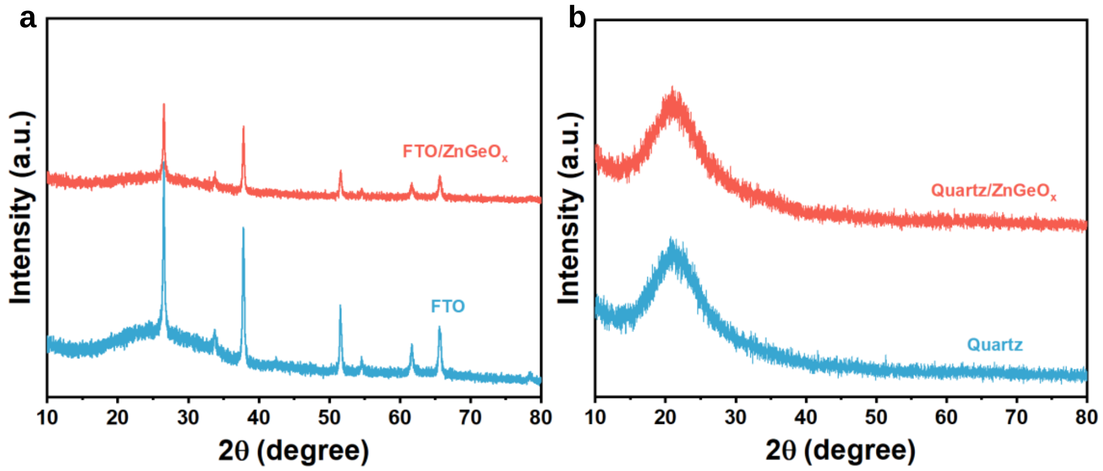

Supplementary Figure 1. XRD patterns of the ALD-$ZnGeO_x$ films deposited on FTO substrate (a) and quartz substrate (b).

Question 4

On line 150, calculating the thickness of the $ZnGeO_x$ film by measuring cross-sectional thickness is very inaccurate, especially on such a rough Cu_2O surface. Such a statement is unscientific. Using a step profiler or ellipsometry would yield more precise and accurate results.

We thank the reviewer for checking our work in detail and pointing out this. The reviewer is correct. We have corrected this inaccurate description. In order to test the growth rate of $ZnGeO_x$ films, the $ZnGeO_x$ films were deposited on clean Si substrates. The $ZnGeO_x$ films with different thickness were obtained by changing the number of super cycles of deposition. A step profiler is used to measure the specific thickness of the $ZnGeO_x$ film (Supplementary Figure 3). As shown in Supplementary Figure 4, the growth rate of the $ZnGeO_x$ film was about 0.55 nm/super cycle. Therefore, forty super cycles of atomic layer deposition result in a $ZnGeO_x$ film of approximately 22 nm in thickness.

Revised manuscript:

"*In order to obtain the thickness of the $ZnGeO_x$ film, we deposited the $ZnGeO_x$ film with different numbers of super cycles on clean Si substrates. As shown in Figure S4, the growth rate of $ZnGeO_x$ films measured by a step profiler is ~0.55 nm/super-cycle. Therefore, forty super cycles of atomic layer deposition result in a $ZnGeO_x$ film of approximately 22 nm in thickness.*"

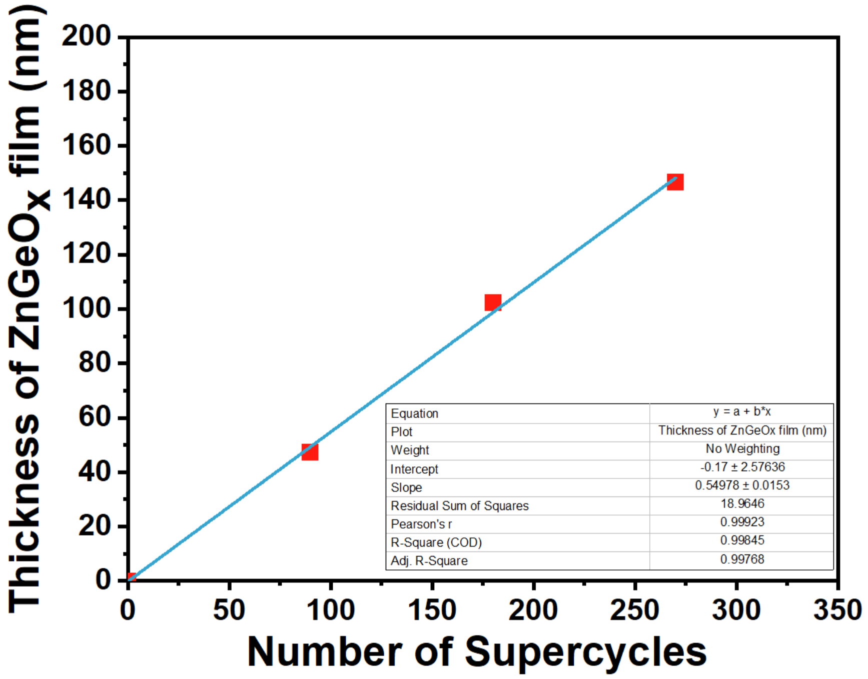

Supplementary Figure 4. ALD-$ZnGeO_x$ growth rate with linear fitting measured using a step profiler. The $ZnGeO_x$ films were deposited on clean Si substrates.

Question 5

On line 152, the step profiler should be shown in Figure S2. In Figure 2c, the marking

for different ALD layers appears arbitrary. It is challenging to observe a clear difference between them (at least in this figure). In contrast, the difference of ALD layers in Fig 2b is more apparent.

We thank the reviewer for pointing out this. The reviewer is correct. In fact, due to the similar atomic numbers of Ti (22), Zn (30), Ge (32), Ga (31) and Cu (29) elements, the contrast in the STEM images is not obvious. Therefore, it is challenging to observe a clear difference between every ALD layer by STEM images. According to the reviewer's suggestion, we re-labeled different ALD layers in Figure 2b in the revised manuscript. The analysis results of element mapping images (Figure 2e-k) and the line profiles for different elements across every interface (Supplementary Figure 5) can also prove that different ALD layers are stratified rather than blended. Moreover, the original data for the thickness of ALD-$ZnGeO_x$ measured using a step profiler were also supplemented in Supplementary Figure 3 of the supporting information in the revised manuscript.

Revised manuscript:
"*From the elemental mapping images, Au, Cu, Ga, Zn/Ge, and Ti are detected from bottom to top in the Cu_2O photocathode, indicating that the device structure is stratified. This stratified structure can also be proved by the line profiles for Ti, Zn, Ge, Ga and Cu elements across the TiO_2, $ZnGeO_x$, and Ga_2O_3/Cu_2O interfaces (Figure S5).*"

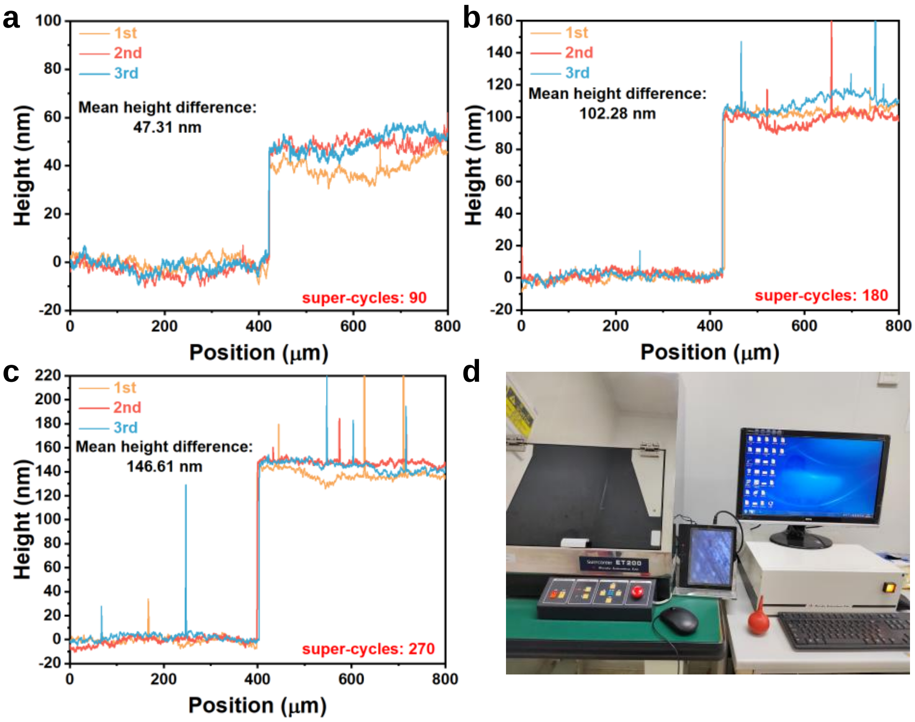

Supplementary Figure 3. (a-c) The original data for the thickness of ALD-$ZnGeO_x$ measured using a step profiler. (d) Photo of the step profiler. The $ZnGeO_x$ films were deposited on clean Si substrates. The thickness of each $ZnGeO_x$ sample was tested three times and its average was taken to fit the growth rate.

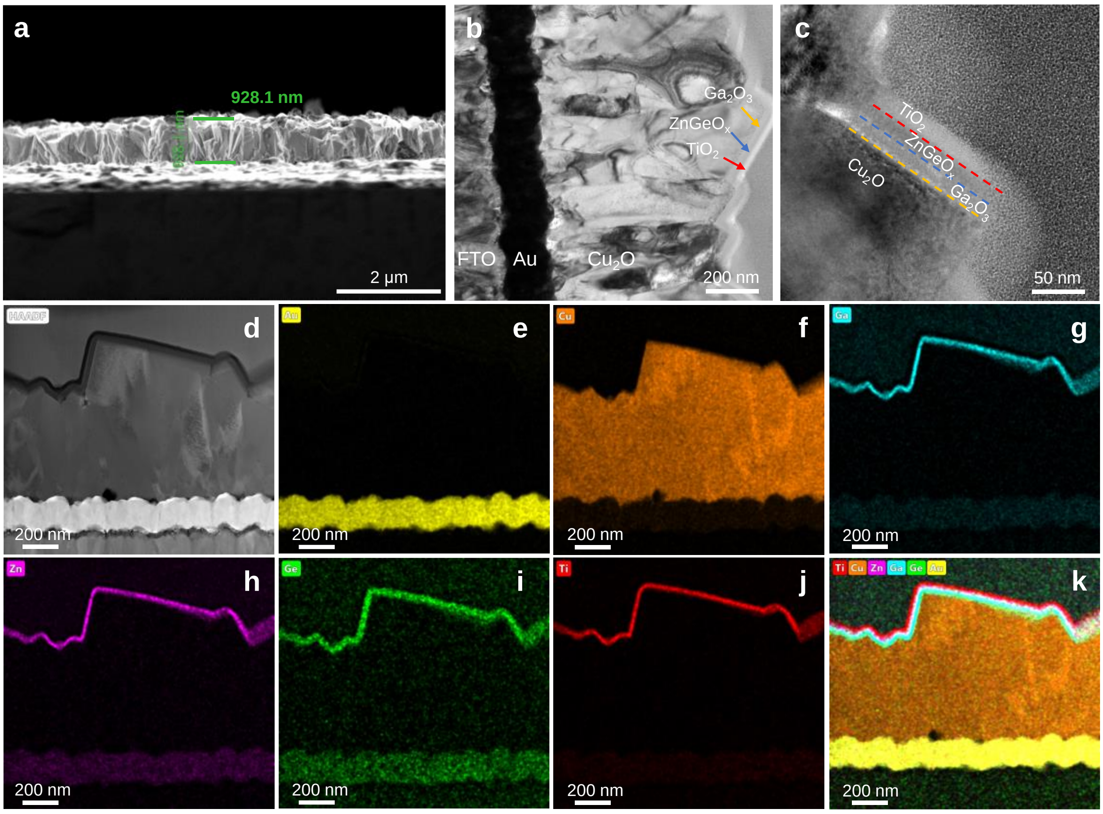

Figure 2. SEM and TEM analysis of the $Cu_2O/Ga_2O_3/ZnGeO_x/TiO_2$ photocathode. (a) Cross-sectional SEM image. (b) The cross-sectional STEM bright-field image. (c) The cross-sectional STEM bright-field image of the top of the $Cu_2O/Ga_2O_3/ZnGeO_x/TiO_2$ photocathode. (d) The STEM high-angle annular dark-field image. (e-j) The corresponding elemental mapping images of Au, Cu, Ga, Zn, Ge and Ti, respectively. (k) Combined elemental mapping image of Au, Cu, Ga, Zn, Ge and Ti.

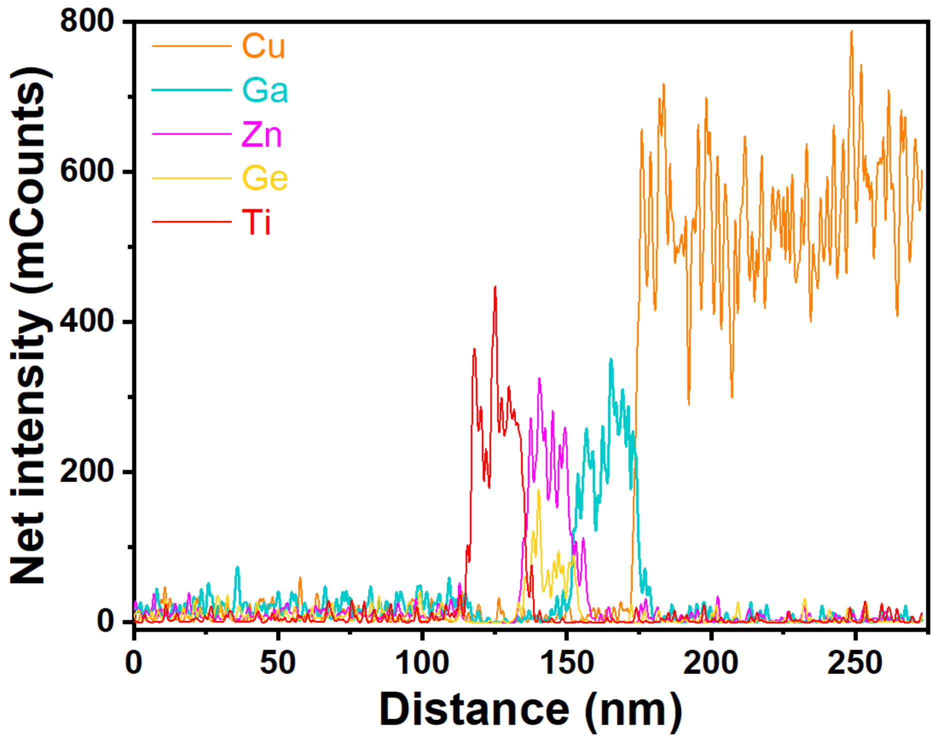

Supplementary Figure 5. The line profiles for Ti, Zn, Ge, Ga and Cu elements across the TiO_2, $ZnGeO_x$ and Ga_2O_3/Cu_2O interfaces.

Question 6

In Figure 4b, the band bending of the $ZnGeO_x$ and TiO_2 layers is misrepresented. For example, at the Ga_2O_3/TiO_2 interface, since the Fermi level of TiO_2 is lower than the H^+/H_2 energy level (some papers claim a higher Fermi level), the TiO_2 should exhibit downward band bending.

We thank the reviewer for checking our work in detail and pointing out this, which helps to improve the quality of the manuscript. The reviewer is correct. The band bending of TiO_2 was wrong in our original manuscript. In fact, the energy band bending should be upward from the interface to the TiO_2 bulk when Ga_2O_3 or $ZnGeO_x$ contacts with TiO_2. And at the interface of photocathode/electrolyte, the TiO_2 should also exhibit downward band bending. We have corrected this error in the revised manuscript.

Revised manuscript:

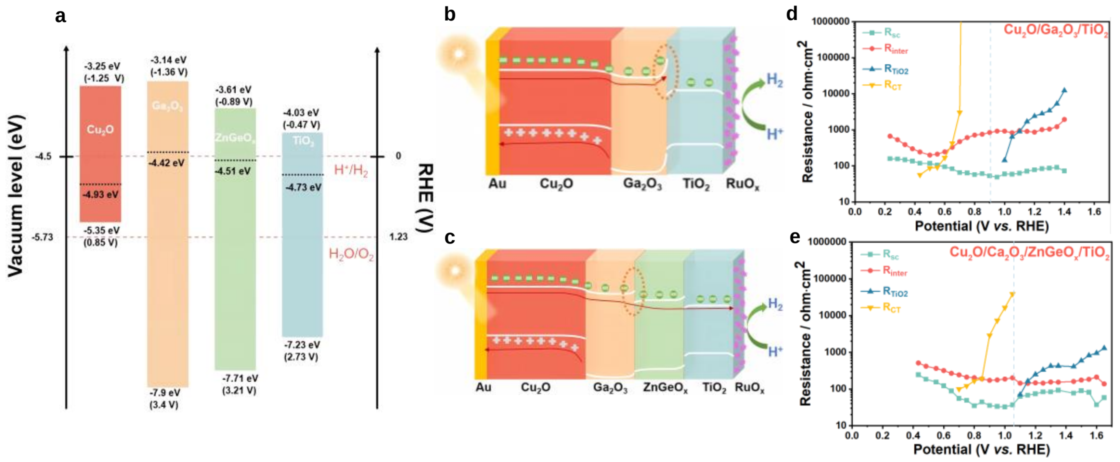

Figure 4. (a) Energy diagrams of Cu_2O, Ga_2O_3, $ZnGeO_x$, and TiO_2 (from left to right) derived from Kelvin probe force microscopy (KPFM), XPS valence band spectra and the Tauc plots. (b) Schematic diagram of the photogenerated carrier transport behavior in the $Cu_2O/Ga_2O_3/TiO_2/RuO_x$ photocathode and (c) the $Cu_2O/Ga_2O_3/ZnGeO_x/TiO_2/RuO_x$ photocathode under illumination, respectively. (d) Resistances from the EIS fitting of the $Cu_2O/Ga_2O_3/TiO_2/RuO_x$ photocathode and (e) the $Cu_2O/Ga_2O_3/ZnGeO_x/TiO_2/RuO_x$ photocathode under illumination, respectively.

Question7

The fitting and extraction of R_{ct}, R_{sc}, R_{tio2}, and R_{inter} from the EIS data should be clearly described in the supporting information.

According to the reviewer's suggestion, the characteristic frequency of every resistance element has been supplemented in the revised manuscript (Supplementary Table 1).

Revised manuscript:

"Based on the number of processes observed in the Nyquist plot, a corresponding number of simple resistors and capacitors are used to fit the EIS spectra. Resistors and capacitors corresponding to the same process are connected in parallel, while elements of different processes are connected in series (Figure S15a). To account for non-ideality of the capacitors constant phase elements, CPEs, have been used (with the exponent accounting for the ideality of the CPE not going below 0.8). This fitting method could result in a reduced picture of the photophysical processes. However, the determined resistances and their dependence on the applied potential still enable one to draw valuable conclusions on the operation of the system and the assigning of the resistances to certain photophysical or electrochemical process. Generally, we can assume that photogenerated charge carriers are subjected to a recombination process inside the space charge region of the photoabsorber. This process normally takes place in the μs range for sufficiently efficiently devices and is therefore situated in the HF region of the EIS measurements (MHz down to KHz). The recombination resistance associated to this process will increase when the recombination current is suppressed.

Therefore, before the bias reached HER onset potential or the recombination was suppressed by a strong band bending, the recombination resistance associated to this process is normally constant. As for the slow process associated with the electron charge transfer into the electrolyte, the corresponding resistance in EIS measurements appears in the mHz to Hz range.[3] The characteristic frequency of every resistance element is displayed in Supplementary Table 1."

Supplementary Table 1. Frequency regions of the detected resistance under illumination.

Resistance	Frequency range
R_{SC}	100 kHz-10 kHz
R_{inter}	10 kHz-40 Hz
R_{TiO2}	1 kHz-mHz
R_{CT}	80 Hz-mHz

Question 8

From Figure 4c, the most significant difference appears to be the R_{ct} value; however, the authors attribute the improvement only to the R_{inter} value, which is not convincing. A direct comparison of the Nyquist plot at potentials between 0.6 and 0.8 V RHE will be more illustrative.

We thank the reviewer for the suggestion. We have added the direct comparison of the Nyquist plots for the $Cu_2O/Ga_2O_3/TiO_2/RuO_x$ photocathode and the $Cu_2O/Ga_2O_3/ZnGeO_x/TiO_2/RuO_x$ photocathode at potentials between 0.6 and 0.85 V in the revised manuscript. The results were presented in Supplementary Figure 16. It is correct that the R_{CT} value of both photocathodes at the same bias is obviously different. However, it is meaningless to compare the absolute values of their R_{CT} at the same bias. The low frequency resistance, R_{CT}, represents the hydrogen-generating charge transfer over the hydrogen evolving catalyst into the electrolyte solution. Before hydrogen evolution, the RuO_x catalyst needs to be reduced to RuO_x^{-1}, that is, Ru(IV) needs to be reduced to Ru(III). R_{CT} is related to the current flow for the reduction step of RuO_x. We think that under the same bias, more photogenerated charges reach the electrolyte through RuO_x, more RuO_x will be reduced faster, and the R_{CT} will be smaller. After the insertion of $ZnGeO_x$, the construction of energy level gradient facilitates photogenerated electrons to the catalyst, which accelerates the reduction of RuO_x. Therefore, it is reasonable that R_{CT} of the $Cu_2O/Ga_2O_3/ZnGeO_x/TiO_2$ photocathode is smaller than that of the $Cu_2O/Ga_2O_3/TiO_2$ photocathode at the same bias. In other words, the adverse barrier at the Ga_2O_3/TiO_2 interface is unfavorable to photogenerated electrons migration, resulting in slow RuO_x reduction and late onset potential. Given that all the photocathodes in this work use the same catalyst RuO_x, that is, having the same overpotential and dynamics. As a result, it is more reasonable to remark the R_{CT} as an indication of the onset potential, rather than an influence factor for onset potential. Therefore, due to the different photovoltage and onset potential for

both the $Cu_2O/Ga_2O_3/TiO_2$ photocathode and the $Cu_2O/Ga_2O_3/ZnGeO_x/TiO_2$ photocathode, it is meaningless to compare the absolute values of their R_{CT} at the same bias.

Revised manuscript:

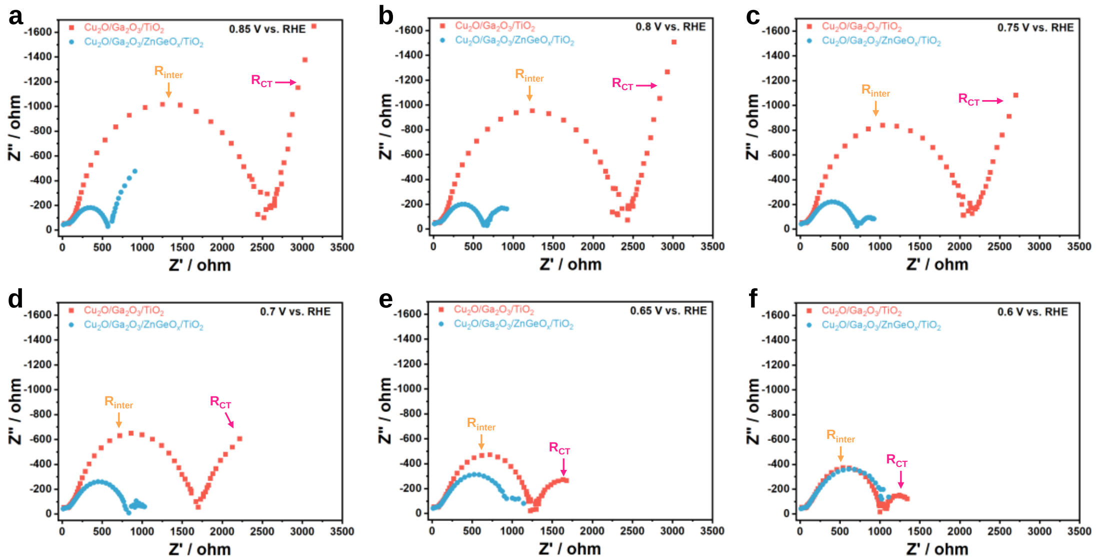

Supplementary Figure 16. A direct comparison of the Nyquist plots the $Cu_2O/Ga_2O_3/TiO_2$ photocathode and the $Cu_2O/Ga_2O_3/ZnGeO_x/TiO_2$ photocathode at potentials between 0.6 and 0.85 V RHE under illumination (light intensity of ~120 W m^{-2}).

Question 9

The authors should provide a clearer explanation of the EIS data. Please ensure that all the above-mentioned issues are addressed to enhance the manuscript's quality and credibility.

According to the reviewer's suggestion, we have added some revisions in the revised manuscript. We have supplemented the descriptions of the fitting and extraction of R_{CT}, R_{SC}, R_{TiO2}, and R_{inter} from the EIS data in the revised supporting information. The characteristic frequency of every resistance element was also supplemented. In addition, for easy understanding of the EIS data, we also added some supplementary descriptions about R_{CT} and R_{SC}. The corresponding fitting resistance element of the EIS semicircles was also marked in the Supplementary Figure 15. We thank the reviewer for the good suggestion and comments. Hopefully, our manuscript provides a clear explanation of the EIS data now.

Revised manuscript:

"R_{CT} is related to the current flow for the reduction step of RuO_x. When the bias is more negative than onset potential for HER, more photogenerated charges reach the electrolyte through RuO_x. RuO_x will be reduced faster, and the R_{CT} will decrease dramatically. For R_{SC}, it is related to the recombination process of the photogenerated

charge carriers. It represents the recombination resistance of the photogenerated electron hole pairs. Before bias reaches onset potential for HER, the electron flow inside the Cu_2O photocathode is negligible. Therefore, the R_{SC} is normally constant. However, once the recombination of photogenerated carriers is suppressed, such as bias exceeding the onset potential, R_{SC} will increase."

"Before H_2 evolution proceeds, their R_{SC} is almost constant, since it is dominated by the recombination process inside the Cu_2O photocathode. The constant R_{SC} implies the negligible electron flow inside the Cu_2O photocathode. However, when the reverse bias exceeds the HER onset potential, both of their R_{SC} values increase gradually, which is caused by the suppressed recombination of photogenerated carriers. The increased R_{SC} indicates the increasing recombination resistance of photogenerated carriers."

REVIEWERS' COMMENTS

Reviewer #1 (Remarks to the Author):

The authors have satisfactorily replied to my comments and the manuscript can now be published.

Reviewer #3 (Remarks to the Author):

The responses are not satisfied. The Cu_2O/TiO_2 based cocatalyst
structure with buffer layers was reported previously,and the performance was not improved in this manuscript especially the saturated photocurrent(see supplementary Table 2) So this manuscript cannot reach the level of NC in terms of novelty or performance improvement. I suggest to be rejected.

Reviewer #4 (Remarks to the Author):

This work gets a higher onset potential but less photocurrent than previous state-of-the-art Cu_2O photocathode. Even though the PEC performance is not the best, with the EIS analysis, this work provides a new strategy to further increases the onset potential of the Cu_2O photocathode.
In the revised version of the manuscript, the author solved most issues I care in last review report. I will recommend this manuscript to be accepted after polishing its languages.
- Some long sentence should be split to shorter one to be more readable.
- The last paragraph is not discussion, but conclusion. The author should check the manuscript carefully to avoid such mistakes.

Point-by-point reply to reviewer comments

We thank all the reviewers for their valuable comments, which we have used to improve our work. Please find below the point-by-point reply to the comments, with the reply in blue color. The revisions made in the revised manuscript are highlighted in yellow color for easy inspection.

REVIEWER COMMENTS

Reviewer #1 (Remarks to the Author):

The authors have satisfactorily replied to my comments and the manuscript can now be published.

We are grateful that Reviewer #1 is satisfied with our response and appreciates the novelty of our work.

Reviewer #3 (Remarks to the Author):

The responses are not satisfied. The Cu_2O/TiO_2 based cocatalyst structure with buffer layers was reported previously, and the performance was not improved in this manuscript especially the saturated photocurrent (see supplementary Table 2). So this manuscript cannot reach the level of NC in terms of novelty or performance improvement. I suggest to be rejected.

We disagree with Reviewer #3. For the Cu_2O photocathode, the Cu_2O/TiO_2 based cocatalyst structure with buffer layer is a universal structure. Previous reports have focused on optimizing the p-n junction interface between Cu_2O and the n-type buffer layer. The photovoltage of Cu_2O photocathodes is improved by optimizing the band alignment between Cu_2O and n-type layers. However, our work presents the concept of dual buffer layers for the first time. And this work reveals the effect of the unfavorable barrier between the buffer layer and the protective layer on the onset potential. This work does not focus on optimizing the band alignment of the p-n junction. This strategy also provides an effective approach to increase the photovoltage of other photoelectrodes with buried junctions.

As for the PEC performance of Cu_2O photocathodes, we admit that Ga_2O_3 can form a good interfacial band alignment with Cu_2O, making the onset potential of the Cu_2O photocathode as high as 1 V (vs. RHE). Nevertheless, the onset potential in our results is 1.07 V, which is still a positive shift of 70 mV compared to the current state-of-the-art Cu_2O photocathodes (Pan et al, Nature Catalysis, 2018, 1, 412-420). It is an important advancement in improving the onset potential of Cu_2O photocathodes. Although the saturated photocurrent density in our results is lower than that of the current state-of-the-art Cu_2O photocathodes (Pan et al, Nature Catalysis, 2018, 1, 412-420), we will further increase the saturated photocurrent density of our samples by improving electrodeposition parameters of Cu_2O films.

Reviewer #4 (Remarks to the Author):

This work gets a higher onset potential but less photocurrent than previous state-of-the-art Cu_2O photocathode. Even though the PEC performance is not the best, with the EIS analysis, this work provides a new strategy to further increases the onset potential of the Cu_2O photocathode. In the revised version of the manuscript, the author solved most issues I care in last review report. I will recommend this manuscript to be accepted after polishing its languages.

We are grateful that Reviewer #4 appreciates the novelty of our work on the new strategy to improve the onset potential of the Cu_2O photocathode.

Question 1

Some long sentence should be split to shorter one to be more readable.

We have carefully polished the language in the revised manuscript. Long sentences that are difficult to understand have been modified into short sentences in the revised manuscript. The revisions made in the revised manuscript are highlighted in yellow color for easy inspection.

Question 2

The last paragraph is not discussion, but conclusion. The author should check the manuscript carefully to avoid such mistakes.

We thank the reviewer for checking our work in detail and pointing out this. According to the format guidelines for *Nature Communications*, we have added a discussion in the Discussion section in the revised manuscript. And we have moved the conclusion to the end of the Results section.

Revised manuscript:

"In this work we have shown that the construction of dual buffer layers in the Cu_2O photocathode can increase the photovoltage to 1.07 V. However, given that the band gap of Cu_2O is 2 eV, the theoretical maximum photovoltage of the Cu_2O photocathode can reach 1.6 V,[49] which leaves a lot of space for further improvement of its photovoltage.

In addition to optimizing the band alignment between the n-type buffer layer and the protective layer, it is important to optimize the band alignment of the p-n junction interface. For transparent n-type layers coupled with Cu_2O, there should be a larger Fermi level difference and smaller conduction band offset relative to Cu_2O. In addition, n-typer layers should have excellent electrical conductivity. Up until now, Ga_2O_3 is still one of the best n-type buffer layers for the Cu_2O photocathode. However, insulator-like Ga_2O_3 has a larger resistance, which is not conductive to the transport of charge carriers. Increasing the carrier densities and conductivity of Ga_2O_3 by heteroatom doping may be an effective solution.[32] In addition, the influence of interfacial defects or

surface states on the photovoltage of the Cu_2O photocathode cannot be ignored. These defects, such as Cu^0 or Cu^{2+}, can narrow the splitting of the quasi-Fermi level and trigger the recombination of hole-electron pairs by restricting the Fermi level of Cu_2O, which eventually decreases the photovoltage.[29, 50] However, the current understanding of defect generation and the specific mechanism affecting the photovoltage is unclear and further investigation is required. The preparation of high-quality single crystal Cu_2O is also worth further development and exploration. Finally, the effect of different crystal groups on the photovoltage of Cu_2O photocathodes is also a very interesting and important research point."
